# MiR-133b targets Sox9 to control pathogenesis and metastasis of breast cancer

**DOI:** 10.1038/s41419-018-0715-6

**Published:** 2018-07-03

**Authors:** Qiu-Yu Wang, Ci-Xiang Zhou, Meng-Na Zhan, Jun Tang, Chen-Long Wang, Cheng-Ning Ma, Ming He, Guo-Qiang Chen, Jian-Rong He, Qian Zhao

**Affiliations:** 1 Department of Pathophysiology, Key Laboratory of Cell Differentiation and Apoptosis of National Ministry of Education, Shanghai Jiao Tong University School of Medicine (SJTU-SM), 200025 Shanghai, China; 2 Institute of Health Sciences, Shanghai Jiao Tong University School of Medicine (SJTU-SM) & Shanghai Institutes for Biological Sciences (SIBS), Chinese Academy of Sciences (CAS), 200025 Shanghai, China; 3 Department of General Surgery, Rui-Jin Hospital, Shanghai Jiao Tong University School of Medicine, 200025 Shanghai, China

## Abstract

The miR-133b, a commonly recognized muscle-specific miRNA, was reported to be deregulated in many kinds of cancers. However, its potential roles in tumorigenesis remain greatly elusive. Herein, we demonstrate that miR-133b is significantly suppressed in human breast cancer specimens, which is reversely correlated to histological grade of the cancer. Ectopic expression of miR-133b suppresses clonogenic ability and metastasis-relevant traits in vitro, as well as carcinogenesis and pulmonary metastasis in vivo. Further studies have identified Sox9, c-MET, and WAVE2 as direct targets of miR-133b, in which Sox9 contributes to all miR-133b-endowed effects including cell proliferation, colony formation, as well as cell migration and invasion in vitro. Moreover, re-expression of Sox9 reverses miR-133b-mediated metastasis suppression in vivo. Taken together, these findings highlight an important role for miR-133b in the regulation of tumorigenesis and metastatic potential of breast cancer and suggest a potential application of miR-133b in cancer treatment.

## Introduction

Breast cancer is one of the most common cancers with >1,300,000 cases and 450,000 deaths each year worldwide^1^. Like many other solid tumors, metastasis is responsible for as much as 90% of breast cancer-related mortality^2^. The invasion–metastasis cascade encompasses multistep process involving local invasion, intravasation, survival in the circulation, extravasation, micrometastasis, colonization, and ultimately outgrowth of secondary tumors^3^. Metastasis is a highly inefficient process, and only a few cells are believed to be able to complete all the steps and develop into macroscopic metastasis^4^. Recent studies suggest that the neoplastic cells within individual tumors are highly heterogeneous and metastases develop from a subset of malignant cells that possess cancer stem cell characteristics^5–7^. During the process of metastasis, tumor-initiating ability would seem to be critical for disseminated cancer cells to seed metastases to vital organs^8,9^.

MicroRNAs are small, non-coding RNAs (18–23 nucleotides) that regulate gene expression by binding to the 3′-untranslated region (UTR) of target mRNAs and trigger translation repression or mRNA cleavage^10^. In mammalian cells, an individual miRNA can regulate dozens of distinct mRNAs and bioinformatics predictions reveal that more than one-third of the protein-coding genes are regulated by miRNAs^11^. MiRNAs play important roles in various biological processes, such as cellular differentiation, proliferation, apoptosis, as well as stem cell maintenance, and their deregulation are associated with the development of various diseases including cancer^12,13^. Recent studies have identified miRNAs that contribute to the development of breast cancer via maintenance of breast stem cells^14^, epithelial-to-mesenchymal transition (EMT)^15^, and mechanisms enabling invasion and metastasis^16,17^.

MiR-133b, which participates in myoblast differentiation and myogenic-related diseases, is commonly recognized as a muscle-specific miRNA^18–21^. Recent reports demonstrated that miR-133b also plays crucial roles in other biology processes such as neuron and fat differentiation^22–25^. Furthermore, miR-133b was also reported to be deregulated in many kinds of cancer^26^ and contributes to malignant progression via influencing cellular proliferation^27,28^, apoptosis^29^, and motility^30^. However, the expression and function of miR-133b appear quite different from cancers. For example, high miR-133b expression levels were found to be associated with poor prognosis for progression-free survival with bladder cancer, whereas its low expression levels in tumor tissues were found to be related to poor prognosis for overall survival and positive lymph node metastasis in colorectal cancer^26^. Despite these studies, whether miR-133b is involved in the development of breast cancer remains largely elusive. In this report, we first demonstrate that miR-133b is pathologically downregulated in breast cancer specimens and cell lines, whereas ectopic expression of miR-133b strongly suppresses clonogenic ability and metastasis-relevant traits in human breast cancer cells. Furthermore, miR-133b expression suppressed tumorigenesis, as well as invasion–metastasis cascade in vivo. Our data further decipher the target genes of miR-133b, one of which sox9 is regarded to promote the tumorigenic and metastasis-seeding abilities. Thus, our findings provide valuable clues toward understanding the mechanisms of human breast cancer metastasis and presents an opportunity to develop more effective clinical therapies in the future.

## Materials and methods

### Patients and tissue samples

Breast carcinoma and adjacent normal tissue were collected from the Comprehensive Breast Health Center, Shanghai Rui-Jin Hospital of Shanghai Jiao Tong University School of Medicine at the time of surgery and immediately frozen to −80 °C until use. A total of 38 paired tissues were involved in our study and their histological types were confirmed by hematoxylin and eosin (H&E) staining. Informed consent was obtained from all patients and this study was approved by the research ethnics committee of Shanghai Jiao Tong University School of Medicine.

### Cell lines and cell culture

Human breast cancer cell lines BT474, SK-BR-3, HCC1937, BT549, and MCF-10A were purchased from the cell bank of the Chinese Academy of Sciences (Shanghai, China). Breast cancer cell lines MDA-MB-231, MDA-MB-468, and MDA-MB-453 were provided by Pro. Ming-Yao Liu (East China Normal University, Shanghai, China) and MCF-7 was obtained from American Type Culture Collection (Manassas, VA, USA). MCF-10A was cultured in Dulbecco’s modified Eagle’s medium (DMEM)/F12 (Gibco, Grand Island, NY, USA) supplemented with 10% horse serum (Gibco), 10 μg/ml insulin (I5500, Sigma-Aldrich, St. Louis, Missouri, USA), 20 ng/ml Epidermal Growth Factor (AF-100-15, PeproTech, London, UK), 100 ng/ml cholera toxin (C8052, Sigma-Aldrich), and 0.5 μg/ml hydrocortisone (H0888, Sigma-Aldrich). MCF-7 was cultured in DMEM with 10% fetal bovine serum (FBS; Hyclone, Logan, Utah, USA) and 10 μg/ml insulin. MDA-MB-231, MDA-MB-468, and MDA-MB-453 were cultured in Leibovitz L-15 medium (Gibco) with 10% FBS. BT549, BT474, and HCC1937 were cultured in RPMI-1640 (Hyclone) with 10% FBS. The breast cancer cell line MDA-MB-231-luc-D3H2LN (Xenogen Alameda, CA) stably expressing firefly luciferase was maintained in MEM/EBSS (Hyclone) supplemented with 10% FBS, 1% non-essential amino acids (Hyclone), and 1% sodium pyruvate (Hyclone). All cells were grown in a humidified atmosphere of 5% CO_2_ and 95% air, except for MDA-MB-231, MDA-MB-453, and MDA-MB-468, which were fostered in 100% air at 37 °C.

### Real-time quantitative RT-PCR

Total RNA was extracted using Trizol reagents (Invitrogen, Carlsbad, CA, USA) according  to the manufacturer’s protocol. RNA was treated with DNase (Promega, Madison, WI, USA). Quantitative stem-loop reverse transcription polymerase chain reaction (stem-loop RT-PCR) was applied to measure the abundance of miR-133b. According to the method, mature miR-133b and U6 snRNA were transcribed with ImProm-II^TM^ Reverse Transcription System (Promega) using Stem-loop RT primer miR-133b-RT and random primers of nonadeoxyribonucleotide. Real-time PCRs were performed with SYBR Green PCR Master Mixture Reagents (Applied Biosystems, Foster City, CA, USA) on the ABI PRISM 7900 system (Applied Biosystems). U6 was used as endogenous control for miR-133b expression analysis and the relative expression levels were evaluated using the 2^-∆∆Ct^ method. Primers used in this study are summarized in Supplemental Table S1.

### MiRNA in situ hybridization

To visualize miR-133b expression in breast tissue, breast cancer microarray section BR2411 (US Biomax Inc, Rockville, MD) were treated and hybridized with DIG-labeled miR-133b probes (Exiqon, Copenhagen, Denmark) overnight at 42 °C. Sections were processed further and incubated with a mouse antidigoxin antibody followed by streptavidin–biotin–peroxidase complex.

### Plasmid construction and generation of stable cell lines

To stably express miR-133b in MDA-MB-231-luc-D3H2LN cells, the 413-bp genomic segment containing the mature miR-133b sequence was amplified and cloned into the PLVX-IRES-ZsGreen vector. Then, the PLVX-miR-133b vector and the control vector with the packaging plasmid pCMV∆8.91 and pMD.G were transfected into HEK293T cells to product lentivirus. MDA-MB-231-luc-D3H2LN cells were transduced with lentivirus and sorted by flow cytometry to obtain stable cell lines. For proving that the predicted target genes are regulated by miR-133b, the 3′-UTR of potential targets were cloned into the downstream of the Renilla luciferase gene in the psiCHECK^TM^-2 vector (Promega). Besides, mutant vectors containing four mutated bases on the predicted binding sites were constructed using the site-directed mutagenesis kit (Stratagene, La Jolla, CA). For WAVE2 and Sox9 overexpression, the complementary DNA of WAVE2 and Sox9 were cloned into the multiple cloning sites of pcDNA3.1 vector and pMSCV puro vector respectively. To obtain stable expression of Sox9 cells, MDA-MB-231-luc-D3H2LN (termed as MDA-MB-231-luc) cells were transduced with the pMSCV-Sox9 retrovirus, and then selected with 1 μg/ml puromycin for 2 days. The selected cells were transfected with miR-133b mimics or infected with PLVX-miR-133b lentivirus for target gene rescue assays. Primers for plasmid construction are shown in Supplementary Table 1.

### RNA oligonucleotide transfection

Both miR-133b and negative control (NC) were synthesized by Genepharma (Shanghai, China), comprised of RNA duplexes with the following sequence orientated from 5′–3′, miR-133b: UUUGGUCCCCUUCAACCAGCUA/GCUGGUU-GAAGGGGACCAAAUU, NC: UUCUCCGAACGUGUCACGUTT/ACGUGACA-CGUUCGGAGAATT. Small interfering RNA duplex oligonucleotides targeting Sox9 mRNA (5′-GCAGCGACGUCAUCUCCAA-3′) and WAVE2 mRNA (5′-GAAAGAUAAUCCAAAUCGA-3′) were synthesized by Ribobio (Guangzhou, China). RNA oligonucleotide was transfected with Lipofecamine 2000 reagent (Invitrogen) at the concentration of 100 nM.

### Cell proliferation and colony formation assays

Cell proliferation kinetics were assayed by seeding 4 × 10^4^ cells per well in six-well plates. After trypsinization, total cell number and cell viability were assessed by Vi-Cell XR Cell Viability Analyzer (Beckman Coulter) every 2 days. To evaluate cell clonogenic ability, 300 cells were seeded in six-well plates for 12 days to form visible colonies, which were then fixed with cold methanol, stained with 1% crystal violet (Sigma, St Louis, Mo, USA) and counted.

### Anchorage-independent growth analysis

MDA-MB-231-luc-D3H2LN stably expressing miR-133b and the control cells were trypsinized and suspended in complete medium with 0.4% agar (Bio-Rad, Hercules, CA). The cell and agar mixture was plated on a 0.6% agar base layer with complete medium at the density of 1.0 × 10^3^ cells per well in six-well plate. For this assay, cells were cultured in soft agar medium for 21 days, and then formed colonies containing >50 cells per well were quantified.

### Cell migration and invasion assays

For migration assay, 5.0 × 10^4^ cells were plated on the membranes with 8.0 μm pores (Corning, USA); For invasion assays, 1.0 × 10^5^ cells were seeded in the chambers with matrigel-coated (BD Biosciences, San Jose, CA, USA). Cells were resuspended in serum-free medium and translocated toward complete growth medium for 18 h. Cells that adhering to the lower surface of the membrane were fixed with cold methanol and stained with 1% crystal violet. Cell migration ability was also assessed by wound-healing assay. Cells were grown to a confluent monolayer and were scraped by a sterile tip to create an artificial wound. The spread of wound closure was photographed under the microscope after 12 h.

### Luciferase assays

MDA-MB-231 cells were seeded in 24-well plates at the density of 1.0 × 10^5^ cells per well. After 24 h, each well was transiently co-transfected with 100 ng of the indicated wild-type or mutant 3′-UTR psiCHECK-2 plasmid and 60 pmol NC or miR-133b mimics using 1.44 μl Lipofectamine reagent (Invitrogen). Cell lysates were collected 24 h after transfection, then Renilla and firefly luciferase activities were measured with a Dual-Luciferase Reporter System (Promega). The Renilla luciferase activities normalized to firefy luciferase activities was the value of relative luciferase activity.

### Western blot analysis

Cell or tissue lysates were resolved on sodium dodecyl sulfate–polyacrylamide gel electrophoresis gels and transferred to Nitrocellulose membrane (Axygen, CA, USA). After blocking with 5% non-fat milk, the membrane was incubated with primary antibodies against with c-Met (#1996-1, Epitomics, Burlingame, CA, USA), WAVE2 (#3659, Cell Signaling Technology, Danvers, MA, USA), Sox9 (#AB5535, Millipore, Billerica, MA, USA), Vinculin (#4650, Cell Signaling Technology), or β-actin (CP01; Calbiochem, San Diego, CA, USA) overnight at 4 °C and then incubated with horseradish peroxidase-conjugated secondary antibody. The signal was detected in a sensitive digital imaging equipment (ImageQuant LAS 4000 mini, GE Healthcare, Piscataway, NJ, USA) using the ECL detection kit (Millipore). The protein fragments were quantified by densitometry using Quantity One software (Bio-Rad).

### In vivo tumorigenesis and metastasis assays

All research involving animals complied with protocols approved by Shanghai Medical Experimental Animal Care Commission. For in vivo tumorigenesis assays, the indicated number of MDA-MB-231-luc-D3H2LN cells suspended in 50 μl phosphate-buffered saline containing 25% Matrigel were orthotopically transplanted into the mammary fat of 5-week-old BALB/c female nude mice. The tumor incidence was measured 3 months post injection and tumor growth rate was monitored by measuring tumor diameters (long and short diameters) every 3 days since 18 days post injection. The tumor volume was calculated as 1/6[π × long diameter × (short diameter)^2^]. When mice were killed, tumors were isolated and weighed. For lung metastasis assays, 6-week-old SCID/Beige mice were injected with 2 × 10^5^ MDA-MB-231-luc-D3H2LN cells via the tail vein. After 2 weeks, the lung metastasis burden was monitored weekly by bioluminescence imaging (BLI) captured with a charge-coupled device camera (IVIS; Xenogen). Four weeks later, all the mice were killed under anesthesia. The lungs were weighed and fixed in 4% polyoxymethylene for H&E staining analysis.

### Immunohistochemistry

To visualize cell proliferation, detection of Ki67 was performed on 4 μm paraffin sections of MDA-MB-231-luc-D3H2LN cell xenografts in nude mice. All nuclei with homogeneous staining were taken as Ki67-positive cells. The Ki67-positive cells were quantified in four randomly selected fields for each tumor sections. We separately counted Ki67-positive cells and total cells in each selected fields and their ratio was considered as the percentage of Ki67-positive cells. For lung metastases evaluation, H&E staining was performed on sections from embedded lung samples.

### Statistical analysis

All data are presented as the mean ± SD; groups were compared using two-tailed Student’s *t*-test. *p*-Values < 0.05 were considered significant.

## Results

### MiR-133b expression is downregulated in breast cancer

**Fig. 1:**
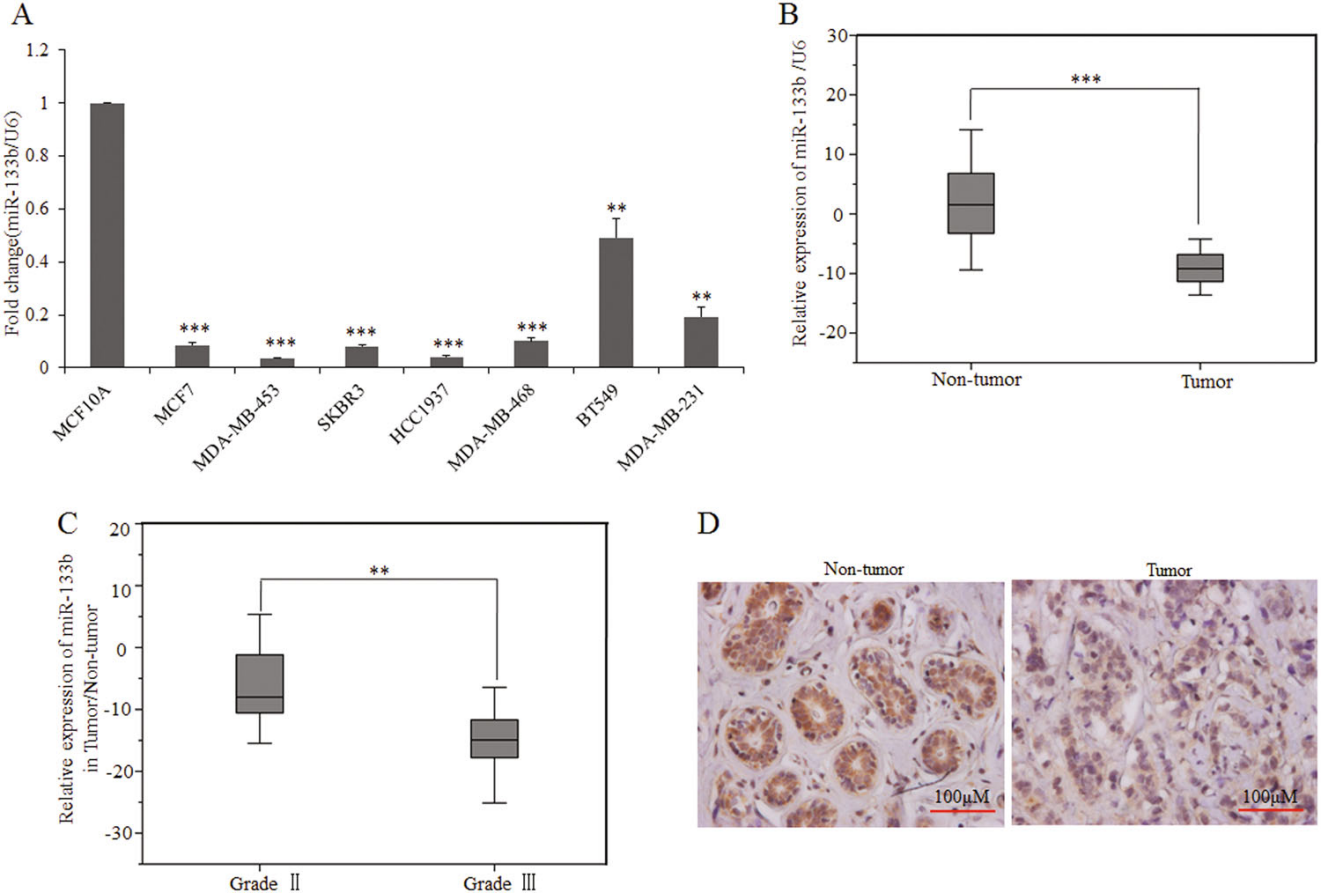
Expression of miR-133b in breast cancer cell lines and specimens. **a** Quantitative RT-PCR analysis of miR-133b expression in eight breast cell lines. The ratio of miR-133b expression levels in breast cancer cell lines to  that in MCF-10A is indicated by a column. **b** Comparison of miR-133b abundance in 38 paired tumor and adjacent non-tumor tissues. The relative expression of miR-133b normalized to the internal control U6 is shown as box plot presentation (*p* < 0.001, independent *t*-test). **c** Expression level of mature miR-133b in Grade II and III breast cancer tissues. The vertical axis presents the relative expression level of miR-133b in tumor tissues normalized to the adjacent non-tumor tissues (*p* < 0.01, independent *t*-test; Grade II, *n* = 16; Grade III, *n* = 18). **d** In situ hybridization of miR-133b in breast cancer tissues and adjacent normal breast tissues on a TMA, which included 24 human samples of breast cancer (scale bars, 100 μm). Data are presented as mean ± S.D. The symbols ** and *** denote significant statistical difference of *p* < 0.01 and *p* < 0.001, respectively, by a two-tailed Student’s *t*-test

We first evaluated the expression of miR-133b in human mammary cell lines by the quantitative RT-PCR analysis. The results showed that, compared with an immortalized normal breast epithelial cell line, the MCF-10A cell, all seven breast cancer cell lines tested presented significantly lower miR-133b level (Fig. 1a). Thus, we investigated miR-133b expression in tumor tissues and paired normal tissues from 38 breast cancer patients. As expected, the expression of miR-133b was downregulated in 35 out of 38 tumor tissues compared with the paired normal tissues (*p* < 0.001, Fig. 1b and Supplementary Table S2). Furthermore, clinic pathological analysis revealed that miR-133b expression level was reversely correlated to tumor grade (*p* < 0.01), with lower expression in grade III (higher degree of malignancy) (Fig. 1c and Supplementary Table S3).

To further describe miR-133b expression levels within mammary epithelial cells in the context of mammary architecture, we applied in situ hybridization with a miR-133b-locked nucleic acid (LNA) probe on breast cancer microarray sections. In accordance with the quantitative RT-PCR analysis (Fig. 1b), strong positive expression of miR-133b was observed in adjacent normal breast tissue while weakly positive expression of miR-133b in infiltrating ductal carcinoma (Fig. 1d and Supplementary Fig. S1). These results indicate that the reduced miR-133b expression was a frequent event in human breast cancer cells and tissues.

### Exogenous overexpression of miR-133b inhibits clonogenic ability and metastasis-relevant traits in vitro

**Fig. 2:**
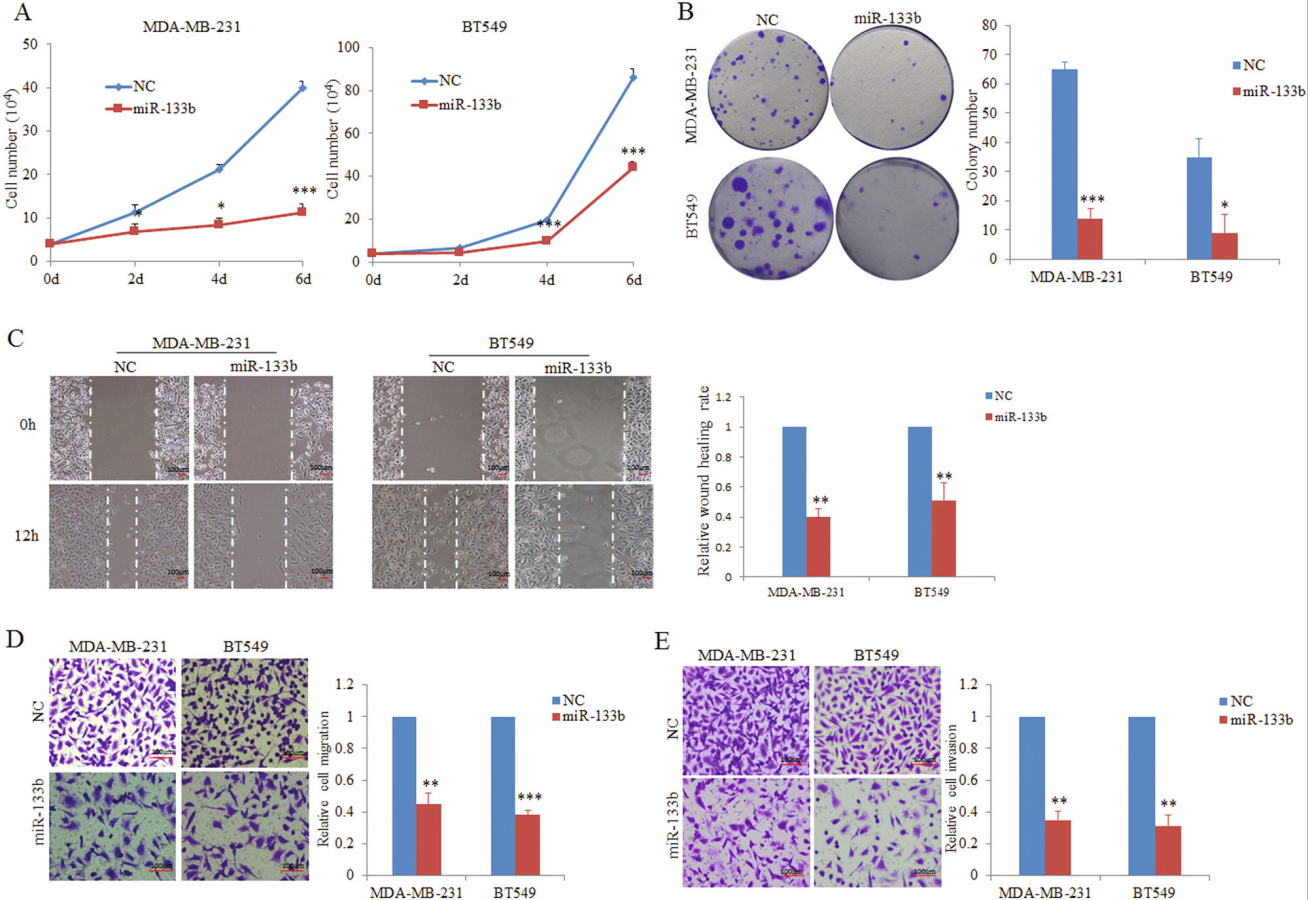
Overexpression of miR-133b suppresses colonigenic ability and metastasis-relevant traits of breast cancer cell lines in vitro. **a** MiR-133b overexpression suppresses proliferation of MDA-MB-231 and BT549 cells. MDA-MB-231 and BT549 cells transfected with 100 nM miR-133b and NC mimics respectively were seeded in six-well plates. Total cell number and cell viability were assessed by Vi-Cell XR Cell Viability Analyzer every 2 days after transfection. **b** Influence of miR-133b on colony formation of MDA-MB-231 and BT549 cells. In all, 300 cells were seeded in six-well plates for 12 days to form visible colonies. Representative images are presented (left panel). The number of clones formed was quantified and shown in the right panel. **c** Wound-healing assay of MDA-MB-231 and BT549 cells transfected with NC or miR-133b mimics in each condition were shown. A scratch wound was made and images from five different fields were taken. Representative pictures of one field at the beginning (*t* = 0 h) and at the end of the recording (*t* = 12 h) were shown (left panel). Cell migration was quantitated as percentage of would-healed area (right panel). **d**, **e** Migration and Invasion assays of MDA-MB-231 and BT549 cells transfected with 100 nM NC or miR-133b mimics. Cell migration **d** and invasion **e** of above cells were quantitatively analyzed 18 h after seeding in Transwells. Data are presented as mean ± S.D. The symbol * denotes statistical difference (*p < *0.05), whereas ** and *** represent great significant difference (*p* < 0.01 and *p* < 0.001) by a two-tailed Student’s *t*-test

To decipher the potential roles of clinically decreased miR-133b in the progression of breast cancer, we transiently transfected two breast cancer cell lines MDA-MB-231 and BT549 with miR-133b or NC mimics. Ectopic expression of miR-133b significantly suppressed cell proliferation in vitro (Fig. 2a and Supplementary Fig. S2A) without having influence on the cell viability (Supplementary Fig. S2B and S2C). The miR-133b-expressing cells also exhibited significantly reduced clonogenic ability (Fig. 2b). To investigate the long-term effect of miR-133b in cell proliferation and colony formation, a lentiviral delivery system was used to stably express miR-133b in metastatic human breast tumor cell lines MDA-MB-231-luc cells. Consistently, stably overexpressing miR-133b in MDA-MB-231-luc cells also inhibited cell proliferation and colony formation (Supplementary Fig. S3A-3C). Moreover, anchorage-independent growth in vitro correlates well with tumorigenicity in vivo^31^. Thus, we performed the soft agar assay to examine the anchorage-independent growth. We found that overexpressing miR-133b could significantly attenuate the colony formation ability of MDA-MB-231-luc cells as exhibited decrease in the colony number and size (Supplementary Fig. S3D).

Having demonstrated the correlation of miR-133b expression to the histological grade in breast cancer tissues, we sought to explore whether miR-133b expression is associated with malignant progression of breast cancer. Considering tumor invasion and metastasis are common features of the most aggressive and lethal tumors, we detected the effects of miR-133b overexpression on metastasis-relevant traits in vitro. As expected, in wound-healing and Transwell migration assays, MDA-MB-231 and BT549 cells overexpressing miR-133b displayed a significant reduction in migration compared with controls (Figs. 2c, d). Overexpression of miR-133b also caused a significant reduction in invasive capacity, assessed by Matrigel invasion assays (Fig. 2e). The similar results were observed when stable overexpression of miR-133b in MDA-MB-231-luc cells (Supplementary Fig. S3E). These results show that ectopic expression of miR-133b suppressed clonogenic and metastatic ability of breast cancer cells in vitro.

### MiR-133b suppresses tumorigenesis, as well as invasion-metastasis cascade in vivo

**Fig. 3:**
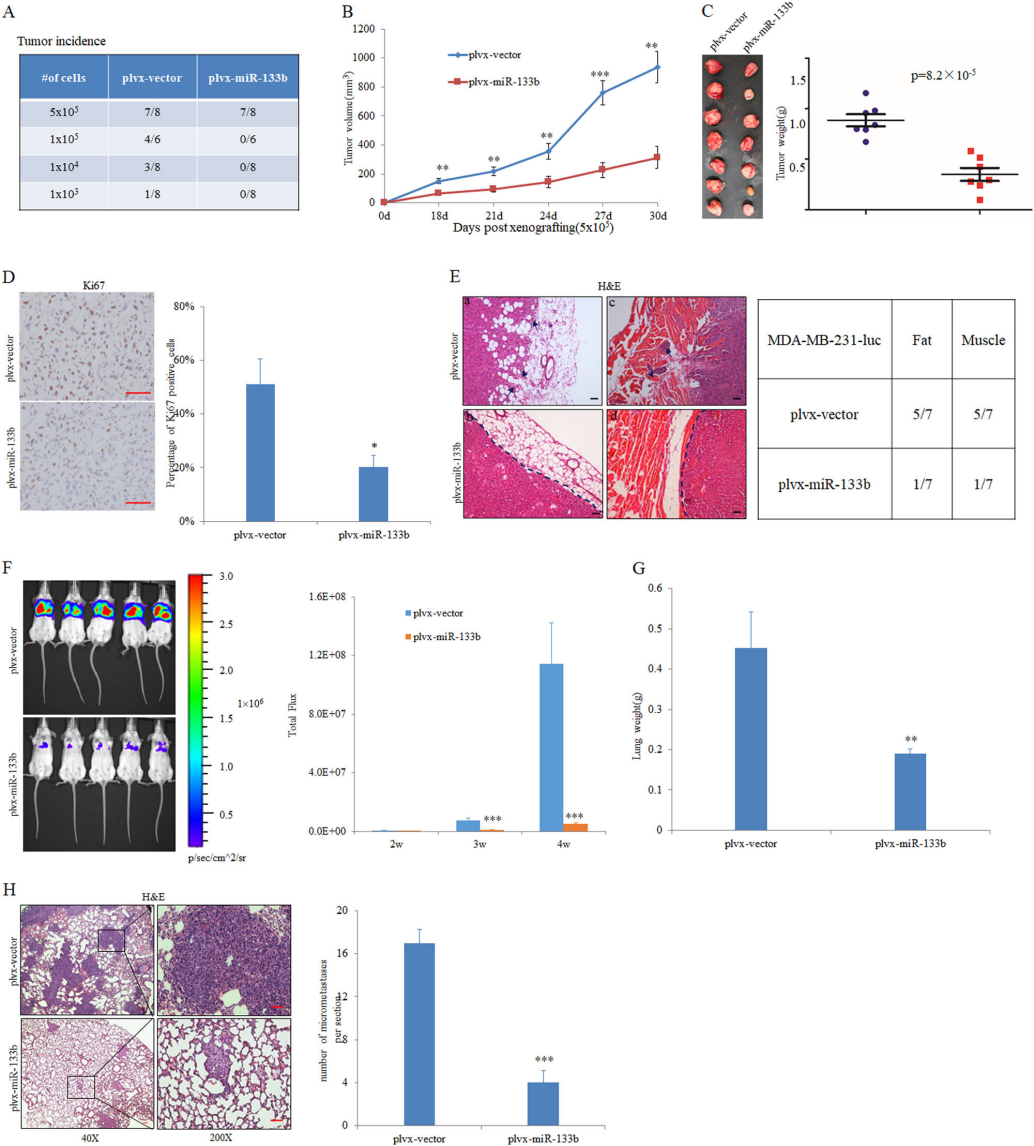
MiR-133b overexpression inhibits tumorigenesis and metastasis in vivo. **a** The tumor incidence of MDA-MB-231-luc cells with or without miR-133b overexpression. Cells were orthotopic injected into the mammary fat pad of mice at the indicated numbers. The tumor incidence was determined 3 months post injection. **b**-**e** In vivo tumorigenesis assays of MDA-MB-231-luc cells with or without miR-133b overexpression. In all, 5 × 10^5^ MDA-MB-231-luc-miR-133b or control cells were injected into the mammary fat pad of mice. **b** Left panel: Tumor volumes of MDA-MB-231-luc xenograft tumors infected with either control or miR-133b expressing vector at different days (*n* = 7 per group). Right panel: relative tumor volume at day 30 after injection of control or miR-133b expressing vector in nude mice. **c** Tumors were separated from the indicated mice and weighted after 30 days post injection. **d** The representative IHC staining images and the percentage of positive Ki67 cells in MDA-MB-231-luc cells-originated tumors at day 30 after injection. Red arrows point to Ki67-positive cells. **e** H&E stain of tissue adjacent to the indicated MDA-MB-231-luc primary tumors 30 days post injection. Arrows: disseminated tumor cells in normal fat (**a**, **b**) and muscle (**c**, **d**). **f**-**h** In vivo metastasis assays of MDA-MB-231-luc cells with or without miR-133b overexpression. Lung metastases were assessed by BLI measurements. The representative BLI images of mice at 4 week after injection are presented at left panel and quantitation of pulmonary metastases are show at right panel (*n* = 5 per group) (**f**). Wet lung weights in tumor-bearing mice were measured (**g**). Representative H&E staining of lungs shows metastatic nodules at ×40 and ×200 magnifications. Visible micrometastases were counted and analyzed (**h**). Data are presented as mean ± S.D. The symbol * denotes statistical difference (*p <* 0.05), whereas ** and *** indicate great significant difference (*p* < 0.01 and *p* < 0.001) following a two-tailed Student’s *t*-test. The scale bar represents 100 μm

In light of the effects of miR-133b on in vitro traits are associated with high-grade malignancy, we explore whether ectopic miR-133b could inhibit tumorigenesis and metastasis in vivo. To this end, we chose MDA-MB-231-luc cells as our cell model of in vivo study because of its highly tumorigenic and metastatic ability. Thus, MDA-MB-231-luc cells stably expressing miR-133b were injected into the mammary fat pad of nude mice. We found that overexpression of miR-133b greatly inhibited the tumor-initiating ability of MDA-MB-231-luc cells. The frequency of primary tumor formed by miR-133b-expressing cells was less than  that of  the control cells through limiting dilution analysis (Fig. 3a). In vivo tumorigenesis assays of MDA-MB-231-luc cells with or without miR-133b overexpression were also conducted. Compared with the control group, the tumor volume and weight from miR-133b-expressing cells were also significantly decreased by 66.58 and 60.59% at day 30 post injection, respectively (Figs. 3b, c). Accordingly, the percentage of Ki67-positive cells in miR-133b-expressing tumors was less than  that of  the control tumors (Fig. 3d). These results showed that ectopic miR-133b attenuate the tumorigenicity of MDA-MB-231-luc cells in vivo.

Besides its dramatic effects on tumor initiation and growth in vivo, ectopic miR-133b expression also effectively suppressed local invasion of the primary tumor. Control primary tumors displayed evidence of local invasion, whereas miR-133b-expressing tumors were well encapsulated and noninvasive (Fig. 3e). To examine whether suppression of tumor-initiating and invasion ability conferred a metastatic disadvantage to miR-133b in vivo, MDA-MB-231-luc cells stably overexpressing miR-133b were transplanted into the SCID/Beige mice via tail vein injection. Bioluminescence imaging (BLI) showed a significant reduction in lung metastases (Fig. 3f). Parallel with BLI results, miR-133b-expressing group showed obvious decrease in lung weights for the less pulmonary metastasis burden compared with the control group (Fig. 3g). Consistently, H&E staining of lungs showed that the number and size of micrometastases were significantly deceased in miR-133b-expressing mice than that of control mice (Fig. 3h). Collectively, these results showed that ectopic miR-133b expression was capable of suppressing invasion and metastasis in vivo.

### MiR-133b directly regulates multi-targets associated with tumor malignancy

To further investigate the molecular mechanism of miR-133b-mediated pathological functions in tumor initiation and progression, we used two mRNA target-predicting algorithms-TargetScan and PicTar to identify the candidate target genes of miR-133b. Totally >200 mRNAs were predicted to be regulated by miR-133b through the analysis. Guided by gene ontology analysis, we selected nine potential targets (*RhoA, CRK, CDC42, MMP14, TGFBR1, WAVE2, COL1A1, Sox9,* and *c-Met*) that were associated with tumorigenesis and metastasis-related functions such as cell proliferation, apoptosis, adhesion, cytoskeletal remodeling, and invasion. Of note, *c-Met* as the target of miR-133b has been reported in colorectal cancer^27^ and was chosen as a positive control in our study.

**Fig. 4:**
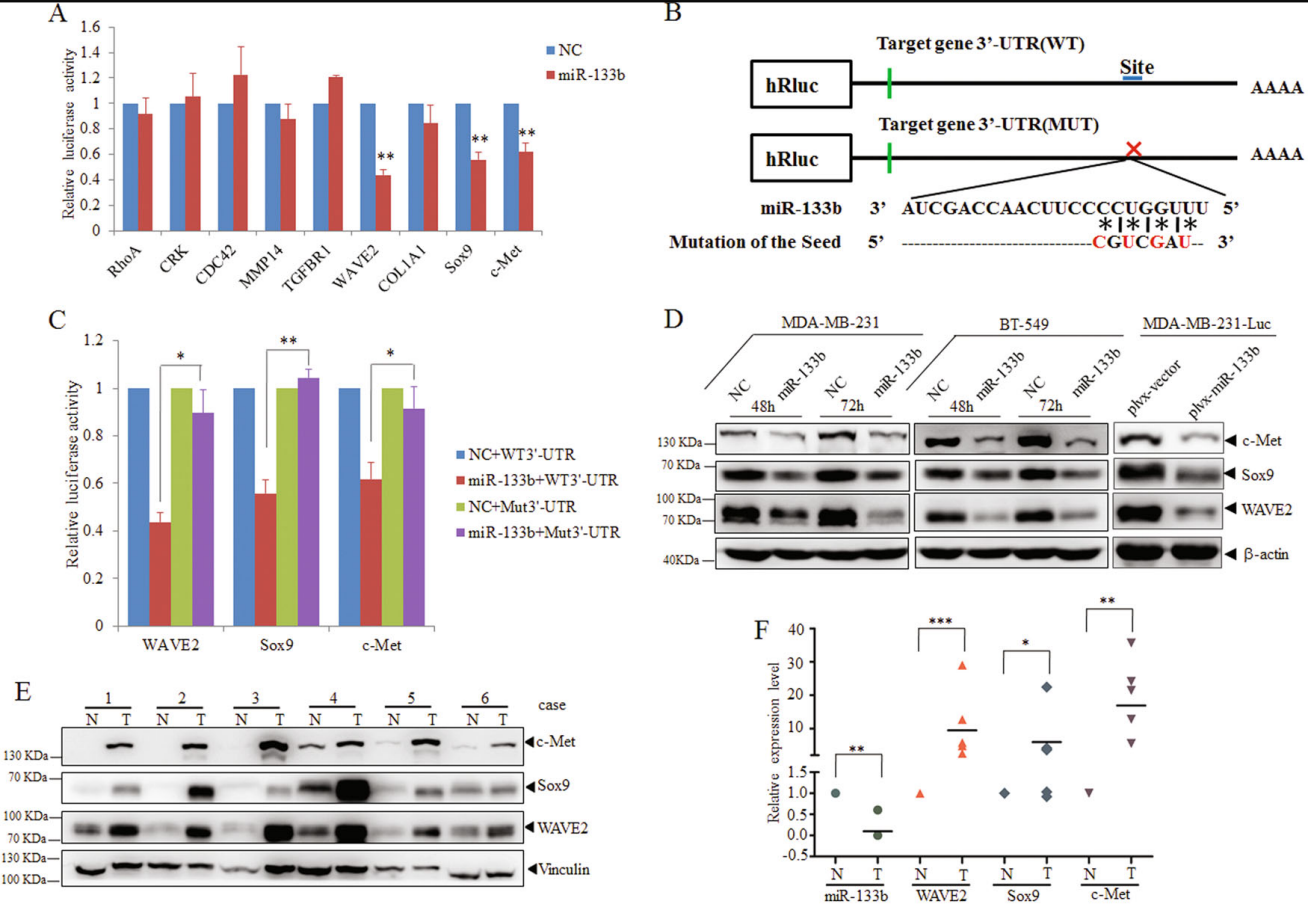
c-Met, Sox9, and WAVE2 are direct targets of miR-133b and are negatively correlated with miR-133b expression in breast cancer. **a** Dual-luciferase assays showing that repression of potential target genes by miR-133b was measured by luciferase activity in MDA-MB-231 cells. The data are mean ± s. e. m. of separate transfections (*n* = 3), and are shown as the ratios of Renilla activity to Firefly activity. **b** A schematic of cloning miR-133b binding sites mutant 3′-UTR of target genes into the dual-luciferase vector. The substitutions of nucleotides within binding sites are shown in red. **c** Relative activity of the luciferase gene fused with the wild-type or mutant 3′-UTR of WAVE2, Sox9, and c-Met genes, respectively. The data are mean ± s. e. m. of separate transfections (*n* = 3), and are shown as the ratio of Renilla activity to Firefly activity. **d** Western blot analysis of c-Met, Sox9, and WAVE2 expression in multi-type breast cancer cell lines with or without miR-133b overexpression. Left two panels: Endogenous WAVE2, Sox9, and c-Met proteins were detected when MDA-MB-231 and BT549 were transfected with NC or miR-133b mimics for 48 and 72 h. Right panel: Immunoblotting of candidate proteins in MDA-MB-231-luc-miR-133b and control cells (plvx-vector). β-Actin serves as internal control. **e** Expression of c-Met, Sox9, and WAVE2 in six paired clinical breast cancer specimens. N and T mean adjacent normal tissue and paired breast carcinoma, respectively. Vinculin serves as internal control. **f** Correlation of miR-133b and target genes in paired clinical breast cancer samples. The expression level of WAVE2/Sox9/c-Met was designated as the ratio of optical intensity between target gene and vinculin in each line, whereas the relative abundance of miR-133b was determined by stem-loop RT-PCR. For miR-133b and WAVE2/Sox9/c-Met, their expression level was assumed as 1 in adjacent normal tissues and corresponding calculated in paired tumor tissues. The symbol * denotes statistical difference (*p* < 0.05), whereas ** and *** represent great significant difference (*p* < 0.01 and *p* < 0.001) by a two-tailed Student’s *t*-test

Based on the above analysis, we cloned the 3′-UTRs of the nine putative miR-133b targets into a dual-luciferase UTR vector-psiCHECK^TM^-2, respectively (Table S1). Luciferase assays in MDA-MB-231 cells revealed that the 3′UTRs of *WAVE2*, *Sox9*, as well as *c-Met* were repressed by miR-133b. Moreover, mutation of the putative miR-133b binding sites in these three 3′-UTRs all relieved the repression caused by miR-133b (Figs. 4b, c). In accordance with the above results, overexpression of miR-133b efficiently reduced endogenous protein level of WAVE2, Sox9 as well as c-Met in MDA-MB-231 and BT549 cells (Fig. 4d). The similar results were observed in MDA-MB-231-luc cells with stably infected miR-133b-expressing PLVX-IRES-ZsGreen vector (Fig. 4d). Furthermore, there is a similar inverse correlation between the expression level of miR-133b and the protein level of WAVE2/Sox9/c-Met in clinical samples (Figs. 4e, f). These data indicated that miR-133b directly regulates endogenous expression of WAVE2, Sox9, as well as c-Met in human breast cancer.

**Fig. 5:**
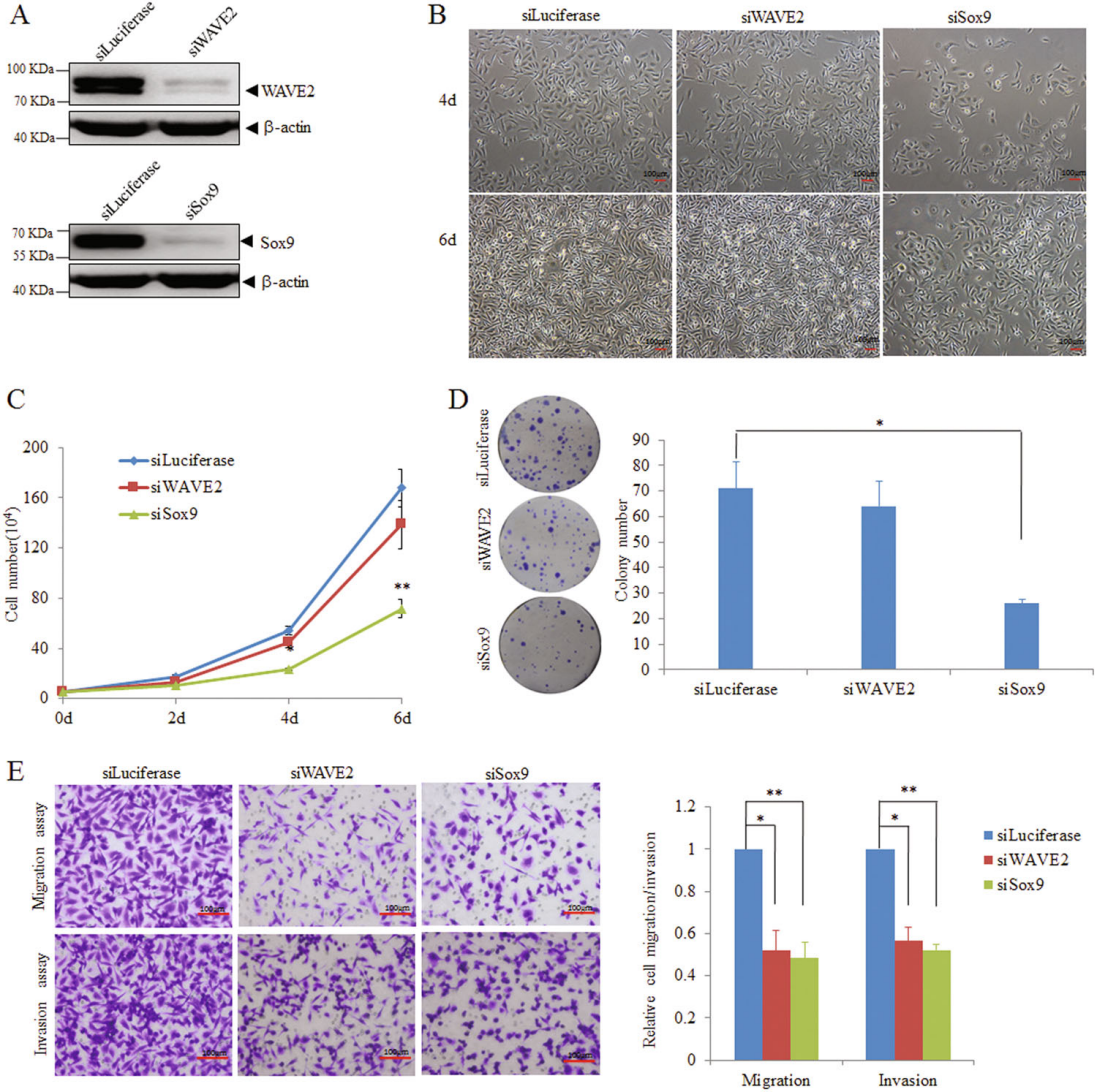
Knockdown of Sox9 imitates miR-133b-mediated phenotypes in MDA-MB-231. **a** Detection of endogenous WAVE2 and Sox9 expression by western blot at 48 h after transfection of 100 nM siWAVE2 or siSox9, respectively, in MDA-MB-231 cells. β-Actin serves as an internal control. **b**, **c** Effects of transfection of siWAVE2 or siSox9 on the proliferation of MDA-MB-231 cells. **b** Morphology of MDA-MB-231 cells at day 4 and day 6 post transfection of siWAVE2 or siSox9 (scale bars, 100 μm). **c** Proliferation curves of MDA-MB-231 cells transfected with siWAVE2 or siSox9. Triplicate wells from each cohort were counted for 6 days. **d** Influence of siWAVE2 or siSox9 on colony formation of MDA-MB-231 cells. Left panel: representative images by colony formation assay. Right panel: the number of clones were counted for each well of six-well plates and shown in the *y* axis. The results were reproducible in three independent experiments. **e** Transwell migration and invasion assays of MDA-MB-231 cells transfected with 100 nM siWAVE2 or siSox9, respectively. The cell migration and invasion were quantitatively analyzed 18 h after seeding in Transwells. Data are presented as mean ± S.D. The symbol * denotes statistical difference (*p* < 0.05), whereas ** represents great significant difference (*p* < 0.01) in a two-tailed Student’s *t*-test. All experiments were repeated independently for three times

To assess the functional contributions of these targets to the phenotypes of miR-133b, we first examined whether their inhibition affected the tumorigenic and metastatic ability of breast cancer cells in vitro. As shown in Figs. 5a, b, transfection of siRNAs against WAVE2 or Sox9 in MDA-MB-231 cells potently reduced the target protein levels respectively without affecting cell viability (Figs. 5a, b). It is worthy to point that knockdown of Sox9 in MDA-MB-231 cells attenuated cell proliferation and colony formation, whereas silencing WAVE2 expression failed to affect these traits (Figs. 5b, c). The similar results were also observed in BT549 cells (Supplementary Fig. S4A-S4C). However, siRNAs against both WAVE2 and Sox9 could reduce cell migration and invasion in MDA-MB-231, as well as BT549 cells (Fig. 5d and Supplementary Fig. S4D). Collectively, inhibition of WAVE2 only suppressed cell migration and invasion, whereas suppression of Sox9 impaired both tumorigenesis and metastasis-relevant phenotypes in vitro.

### Re-expression of Sox9 reverses miR-133b-dependent phenotypes in vitro

**Fig. 6:**
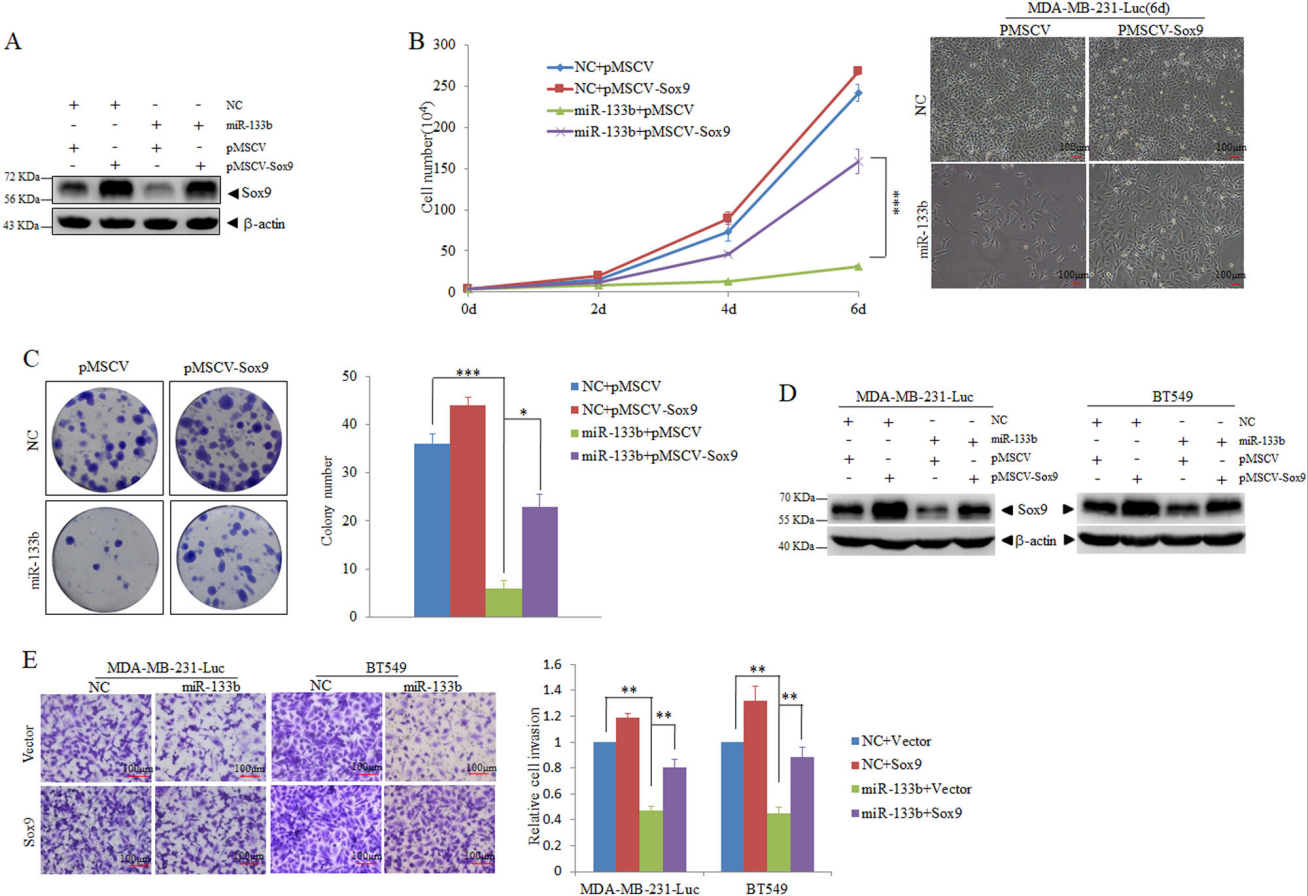
Re-expression of Sox9 reverses miR-133b-dependent phenotypes in vitro. **a** Expression of Sox9 in MDA-MB-231-luc cells infected with pMSCV-Sox9 or control vector after transfection of NC or miR-133b mimics. **b** In vitro proliferation curves of the indicated MDA-MB-231-luc cells transfected with NC or miR-133b mimics. Representative morphology (scale bars, 100 μm) were shown in the right panel. **c** Colony formation assays for the indicated MDA-MB-231-luc cells after transfection of NC or miR-133b mimics. Left panel: Representative images are presented. Right panel: The number of clones were counted for each well of six plates and shown in the *y* axis. **d** Detection of Sox9 expression by western blot in MDA-MB-231-luc or BT549 cells co-transfected with NC or miR-133b mimics together with either pMSCV-Vector or pMSCV-Sox9, respectively. **e** Invasion assays of MDA-MB-231-luc or BT549 cells co-transfected with NC or miR-133b mimics together with either pMSCV-Sox9 or control vector, respectively. Data are presented as mean ± S.D. The symbols * denotes statistical difference (*p* < 0.05), whereas ** and *** represent great significant difference (*p* < 0.01 and *p* < 0.001) by a two-tailed Student’s *t*-test

To further determine whether miR-133b-mediated suppression of cell proliferation and colony formation is ascribable to *Sox9* gene, MDA-MB-231-luc cells infected with pMSCV-Sox9 or control vector were transfected with or without miR-133b mimics. Overexpression of Sox9 efficiently restored Sox9 expression in miR-133b-expressing MDA-MB-231-luc cells (Fig. 6a). As expected, re-expression of Sox9 abrogated cell growth arrest and partially rescued cell colony formation in MDA-MB-231-luc cells transfected with miR-133b, whereas the control vector failed to rescue miR-133-mediated phenotypes (Figs. 6b, c).

To investigate whether re-expression of Sox9 also reverse miR-133b-imposed invasion defect as well, we ectopically expressed Sox9 together with miR-133b in MDA-MB-231-luc and BT549 cells. MDA-MB-231-luc and BT549 cells were co-transfected with NC or miR-133b together with pMSCV-vector or pMSCV-Sox9 for 48 h and Sox9 expression level was validated by western blot (Fig. 6d). The results showed that overexpression of Sox9 abrogated the reduction of invasion ability caused by ectopic miR-133b expression in MDA-MB-231-luc and BT549 cells. Like Sox9, overexpression of WAVE2 partially reverses miR-133b-induced inhibition of invasion in MDA-MB-231-luc and BT549 cells in vitro (Supplementary Fig. S5A and S5B). Collectively, these results suggest that Sox9 contributes to the miR-133b-endowed suppression of tumorigenic and metastatic ability of breast cancer cells, whereas WAVE2 only has effect on the cell motility suppression caused by miR-133b.

### Re-expression of Sox9 partially reverses miR-133b-mediated metastasis suppression in vivo

**Fig. 7:**
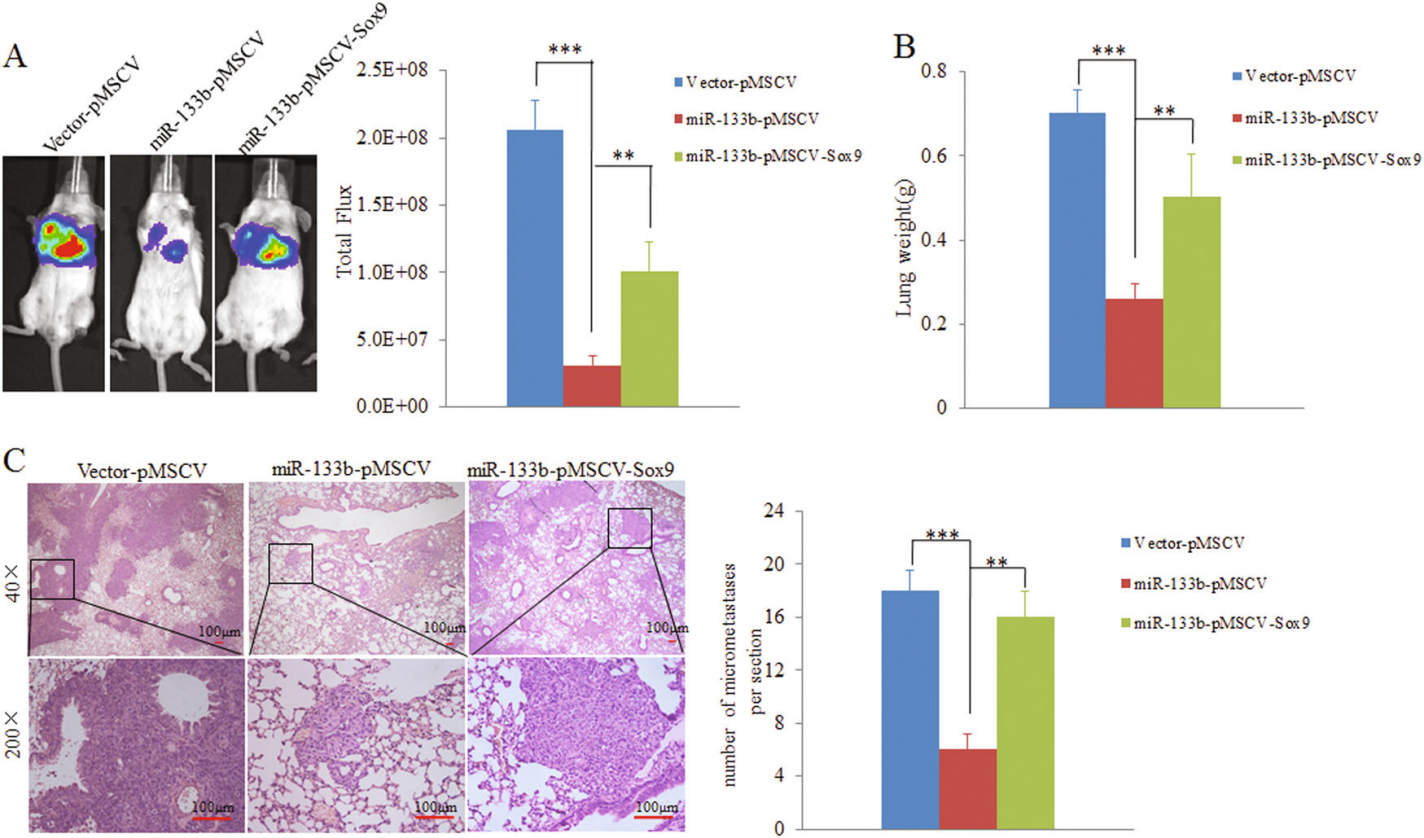
Re-expression of Sox9 relieved miR-133b-mediated metastasis suppression in vivo. **a** In vivo lung metastasis assays of miR-133b-expressing MDA-MB-231-luc cells with or without Sox9 overexpression. Left panel: representative bioluminescence (BLI) images of lung metastases derived from orthotopic injections of control MDA-MB-231-luc cells (Vector-pMSCV), MDA-MB-231-luc-miR-133b cells (miR-133b-pMSCV), and MDA-MB-231-luc-miR-133b-Sox9 cells (miR-133b-pMSCV-Sox9) via the tail vein. Quantitation of pulmonary metastases as assessed by BLI measurements (4 weeks after the injection, *n* = 4 per group). **b** Wet lung weights in tumor-bearing mice were measured. **c** Representative H&E images (scale bars, 100 μm) of lungs showing metastatic nodules. The number of metastatic nodules in the lungs were analyzed (five to six sections were evaluated per lung). Data are presented as mean ± S.D. The symbols ** and *** indicate great significant difference (*p* < 0.01 and *p* < 0.001) following a two-tailed Student’s *t*-test

During the process of metastasis, tumorigenic ability is also critical for disseminated cancer cells to seed metastases in distant organs. So we wonder whether re-expression of Sox9 could rescue the miR-133b-dependent inhibition of metastasis in vivo. Therefore, we stably re-expressed of Sox9 in MDA-MB-231-luc cells that had been infected with either miR-133b-expressing PLVX-IRES-ZsGreen vector or control vector (Supplementary Fig. S6A). As expected, Sox9 did abrogate the suppression of cell proliferation and colony formation induced by miR-133b in vitro (Supplementary Fig. S6B and S6C). Next, MDA-MB-231-luc control cells (vector + pMSCV), MDA-MB-231-luc-miR-133b cells (miR-133b + pMSCV) and MDA-MB-231-luc-miR-133b-Sox9 (miR-133b + pMSCV-Sox9) cells were transplanted, respectively, into the SCID/Beige mice via tail vein injection. Consistent with previous observations, metastases were suppressed in MDA-MB-231-luc-miR-133b cells, and more importantly, re-expressing Sox9 partially rescued metastasis defects of MDA-MDA-MB-231-luc-miR-133b cells in vivo (Fig. 7a). In line with BLI results, lung weights of the miR-133b + pMSCV-Sox9 group were higher than that of the miR-133b + pMSCV group (Fig. 7b). Besides, immunohistochemical (IHC) analysis showed reduced number of lung metastatic nodules in the MDA-MB-231-luc-miR-133b mice compared with control ones (Fig. 7b). Taken together, these data strongly indicate that Sox9 contributes to the miR-133b-endowed ability to inhibit metastasis in vivo.

## Discussion

Accumulating data have pointed that miRNAs play a central regulatory role in the initiation and progression of various cancers^32^. In our study, we demonstrated that a muscle-specific microRNA, miR-133b, was pathologically downregulated in human breast cancer. Moreover, miR-133b levels inversely correlated with tumor grade in a cohort of human breast tumors. Further functional analysis revealed the involvement of miR-133b in the progression of human breast cancer, and overexpression of miR-133b significantly decreased tumor growth and lung colonization in vivo. To the best of our knowledge, this is the first study to explore the role of miR-133b during malignant progression of breast cancer.

Our clinical studies showed that miR-133b was decreased in the resected breast cancer tissues. Recent studies also show that miR-133b is downregulated in colorectal cancer, lung cancer, squamous cell carcinoma, and gastric cancer^27–30^. However, a conflicting report indicated that the elevation of miR-133b has an oncogenic role in the progression of cervical carcinoma^33^. In this study, independent quantitative reverse transcriptase-PCR and in situ hybridization assays were performed to visualize the intact miR-133b expression patterns and confirmed that miR-133b is predominantly expressed in luminal epithelial cells in both ductal and lobular structures of normal breast tissues. Our data showed that miR-133b expression level was inversely correlated to tumor grade in breast cancer. To determine whether the levels of miR-133b are correlated with the clinical outcome in breast cancer patients, we use miRpower, which is a web-tool to validate survival-associated miRNAs utilizing expression data from breast cancer patients. (Website: http://www.kmplot.com/mirpower)^34^. Among the four datasets (METABRIC, TCGA, GSE40267, GSE19783) provided, the METABRIC dataset has long follow-up (median: 94 months), average characteristics (78% Estrogen receptor (ER) positive, 12% human epidermal growth factor receptor 2(HER2) positive), and the largest number of breast cancer patients (1262 patients). Thus we chose this dataset to do the Kaplan–Meier analysis. The Kaplan–Meier analysis showed that breast cancer patients with miR-133b-low tumors had significantly worse overall survival than those with miR-133b-high tumors (Supplementary Fig. S7). This analysis suggests that miR-133b has the potential to be a prognostic marker of breast cancer patients. However, whether the levels of miR-133b are correlated with recurrence of breast cancer patients and could be a predictor of clinical outcome in breast cancer is worthy of further studying.

MiRNAs acts as either oncogenes or tumor-suppressor genes depending on their targets in the particular tissues^35^. In our study, we utilized two bioinformatics algorithms-TargetScan and PicTar to search for the potential targets and finally identify three targets of miR-133b: *c-MET*, *Sox9*, and *WAVE2*. In the colorectal cancer, *c-MET* as one target of miR-133b has been reported to participate in cell proliferation and apoptosis^27^. Here, we focus on the other two novel targets. Loss- and gain-of-function assays demonstrated that WAVE2 was mainly involved in mediating miR-133b-imposed invasion and motility defects, whereas Sox9 contributed to all miR-133b-imposed defects including cell proliferation, colony formation, and invasion ability in vitro. Most importantly, re-expression of Sox9 could reverse miR-133b-mediated metastasis suppression in vivo.

Sox9 is one of the SOX (Sry-related high motility group box) family of transcriptional factors and plays a key role in developmental processes including sex determination, chondrogenesis, neurogenesis, and neural crest development^36–39^. Recent studies demonstrate that Sox9 plays active roles in tumor initiation and progression. For example, Sox9 serves a crucial role in the initiation of pancreatic ductal adenocarcinoma^40^. Sox9 is also reported to be upregulated in brain tumors and basal cell carcinomas. Pediatric and adult high-grade tumors display strong nuclear staining for Sox9 (astrocytic, oligodendroglial and primitive neuroectodermal tumors)^41^. Moreover, gene expression profiling has identified breast cancer subtypes, including an aggressive basal-like (BL) subtype and Sox9 is one of the signature genes that define the BL subgroup of breast cancer. Sox9 protein is expressed at intermediate or high levels in the majority of BL subgroup of breast cancer^42^. Furthermore, coexpression of Sox9 and Slug, which is an important player in epithelial-to-mesenchymal transition, promotes the tumorigenic and metastasis-seeding abilities of human breast cancer cells and is associated with poor patient survival^43^. Consistently, knockdown of Sox9 suppresses the tumor-initiating ability and metastasis of breast cancer. In our study, we used bioinformatics prediction software of TargetScan and PicTar to predict the potential target genes of miR-133b, and finally Sox9 was identified as a functional target gene of miR-133b, which contributed to all miR-133b-imposed defects including cell proliferation, colony formation, and invasion ability in vitro. More importantly, re-expression of Sox9 could reverse miR-133b-mediated metastasis suppression in vivo. These study shows that Sox9 dysfunction contributes to miR-133b biological functions, especially in cancer progression.

WAVE2 belongs to the WAVEs (WASP-family verprolin-homologous proteins) family, which regulate the actin cytoskeleton through activation of Actin-related protein 2 and 3 complex (Arp2/3 complex). As cell motility is regulated by actin cytoskeleton rearrangement and is required for tumor invasion and metastasis, blocking actin polymerization may be an effective strategy to prevent tumor dissemination. It has been demonstrated that WAVEs, especially WAVE2, are essential for invasion and metastasis of melanoma cells, as well as cervical cancer cells^44,45^. The patients with adenocarcinoma of the lung whose cancer cells coexpressed both Arp2 and WAVE2 is correlated with poorer patient outcome, and may be involved in the mechanism of cancer metastasis^46^. Our study demonstrated that miR-133b, through downregulation of its target WAVE2, may contribute to the migration and invasion of the aggressive breast cancer cells like MDA-MB-231 cells.

Collectively, this is the first study to demonstrate that miR-133b is a novel tumor suppressor in breast cancer. Decreased miR-133b participates in the development and progression of human breast cancer by targeting Sox9 that regulates cancer initiation and metastasis. This study suggests that manipulation of miR-133b to impede tumor growth and malignancy may prove to be clinically useful.

## Electronic supplementary material

Table S1

Table S2

Table S3

supplemental legends

Figure S1

Figure S2

Figure S3

Figure S4

Figure S5

Figure S6

Figure S7
