# Supplementary material for: MiR-133b targets Sox9 to control pathogenesis and metastasis of breast cancer
Source: Cell Death Dis. 2018 Jul 3;9(7):752. doi: 10.1038/s41419-018-0715-6 (PMC6030174; doi:10.1038/s41419-018-0715-6)
Supplement: Supplementary file 1 — Table S1 [file 41419_2018_715_MOESM1_ESM.docx]

**Table S1.** Primers for miR-133b quantification, expressing vectors and luciferase reporter plasmids.

| miRNA | Primer | Sequence (5’-3’) |
| --- | --- | --- |
| miR-133b | RT primer | GTCGTATCCAGTGCAGGGTCCGAGGTATTCGCACTGGATACGACTAGCTG |
|  | Forward primer | CTCAGCTTTGGTCCCCTTCAAC |
|  | Reverse primer | GTGCAGGGTCCGAGGT |
| U6 | Forward primer | CGCTTCGGCAGCACATATAC |
|  | Reverse primer | CAGGGGCCATGCTAATCTT |
| miR-133b  (DNA constructs) | Forward primer | CCGGAATTCTTCTGGACAAGGCAAGC |
|  | Reverse primer | CGCGGATCCGCAAAGGCACAGAACAGT |
| WAVE2  (DNA constructs) | Forward primer | TGCTCTAGACCTGAAGCAGGTCCACCA |
|  | Reverse primer | CGGGGTACCGCAGCAGGCAGAAAGAGTTA |
| Sox9  (DNA constructs) | Reverse primer | GGAAGATCTCGGCTTCTCGCCTTTCCC |
|  | Reverse primer | CCGGAATTC GCCATCTTCGCCCTTCGTG |
| RhoA-UTR | Forward primer | CCGCTCGAGAACCTTGCTGCAAGCACA |
|  | Reverse primer | ATTTGCGGCCGCCTGCCTTTATTCTATTAGTAGTTGG |
| CRK-UTR | Forward primer | CCGCTCGAGGGGAGTGTAATGGCAAACG |
|  | Reverse primer | ATTTGCGGCCGCGGCTGGAACGCAGTGGT |
| CDC42-UTR | Forward primer | CCGCTCGAGGTGTCGGCATCATACTAAAAGC |
|  | Reverse primer | ATTTGCGGCCGCGTTTGGGAAGGTGGGAAAG |
| MMP14-UTR | Forward primer | CCGCTCGAGCCTCGCTGGTAAAGGTCAA |
|  | Reverse primer | ATTTGCGGCCGCTCCCTCCACTGGGTCTCAC |
| TGFBR1-UTR | Forward primer | CCGCTCGAGTATCATAGCTCTGAGGCAAGAC |
|  | Reverse primer | ATTTGCGGCCGCAAGGATGGACCAGGGATGT |
| WAVE2-UTR | Forward primer | CCGCTCGAGGGAGAAATCCCAAAGCAGA |
|  | Reverse primer | ATTTGCGGCCGCCGGAGAAGCCAGCAGAA |
| COL1A1-UTR | Forward primer | CCGCTCGAGAACCCGGAAACAGACAAGC |
|  | Reverse primer | ATTTGCGGCCGCGGACGCAGGACAGACTAGGAG |
| Sox9-UTR | Forward primer | CCGCTCGAGCTCTACTCCACCTTCACCTACA |
|  | Reverse primer | ATTTGCGGCCGACAACAGATGACCATACCCT |
| c-Met-UTR | Forward primer | CCGCTCGAGTTTCACTGCCTGACCTTTAA |
|  | Reverse primer | ATTTGCGGCCGCAGGGTGGCTATTCCATCTAG |
| WAVE2-UTR-Mut | Forward primer | TCACCTGTGCTTTCTGCGTCGATAGAATGGGCACCTGCTGGAG |
|  | Reverse primer | CTCCAGCAGGTGCCCATTCTATCGACGCAGAAAGCACAGGTGA |
| Sox9-UTR-Mut | Forward primer | AACCGAAGAAAGAGACGTCGATCCAGAATTCCCTTTGG |
|  | Reverse primer | CCAAAGGGAATTCTGGATCGACGTCTCTTTCTTCGGTT |
| c-Met-UTR-Mut | Forward primer | GGTCCCACAGGCCACCGTCGATTGGCCTGCAGCCGTGA |
|  | Reverse primer | TCACGGCTGCAGGCCAATCGACGGTGGCCTGTGGGACC |
