# Supplementary material for: MiR-133b targets Sox9 to control pathogenesis and metastasis of breast cancer
Source: Cell Death Dis. 2018 Jul 3;9(7):752. doi: 10.1038/s41419-018-0715-6 (PMC6030174; doi:10.1038/s41419-018-0715-6)
Supplement: Supplementary file 2 — Table S2 [file 41419_2018_715_MOESM2_ESM.docx]

**Table S2**. Characteristics of clinical patients with breast cancer.

| **Patient No.** | **Age(year)** | **Tumor size(cm)** | **Grade** | **TNM** | **ER** | **PR** | **HER2** | **MIB-1** | **Metastasis node count** | **Histological subtype** | **Fold repression of miR-133b(log_2_)** |
| --- | --- | --- | --- | --- | --- | --- | --- | --- | --- | --- | --- |
| 1# | 75 | 2 | 2 | I | 0 | 0 | 2 | 40%+ | 0\16 | Ductal | 15.49 |
| 2# | 32 | 5 | 2 | III | 0 | 0 | 3 | 60%+ | 2\25 | Ductal | 17.80 |
| 3# | 56 | 3.5 | 2 | II | 3 | 0 | 3 | 60%+ | 2\9 | Ductal | 13.57 |
| 4# | 54 | 2.5 | 2 | III | 0 | 0 | 3 | 40%+ | 23\30 | Ductal | 2.12 |
| 5# | 50 | 1.5 | 2 | II | 3 | 0 | 2 | 20%+ | 0\13 | Ductal | 18.41 |
| 6# | 54 | 1.5 | 2 | I | 2 | 0 | 3 | 50%+ | 0\20 | Ductal | 10.32 |
| 7# | 61 | 2 | 3 | III | 3 | 3 | 1 | 20%+ | 8\14 | Ductal | 15.25 |
| 8# | 61 | 0.7 | 2 | I | 3 | 2 | 2 | 10%+ | 0\12 | Ductal | -5.36 |
| 9# | 63 | 3 | - | II | 0 | 0 | 2 | 5%+ | 1\15 | Apocrine | 16.29 |
| 10# | 63 | 2.8 | 3 | II | 3 | 3 | 2 | 10%+ | 3\15 | Ductal | -0.60 |
| 11# | 49 | 4 | 2 | II | 1 | 0 | 1 | 5%+ | 0\21 | Ductal | 20.48 |
| 12# | 46 | 1 | 3 | II | 1 | 1 | 2 | 60%+ | 3\21 | Ductal | 10.46 |
| 13# | 56 | 5 | - | III | 1 | 0 | 2 | 10%+ | 14\14 | Lobular and colloid | 23.59 |
| 14# | 38 | 3 | 3 | III | 0 | 0 | 3 | 90%+ | 23\26 | Ductal | 11.69 |
| 15# | 70 | 4 | 2 | II | 2 | 1 | 1 | 10%+ | 0\17 | Ductal | 7.39 |
| 16# | 59 | 2.5 | 3 | II | 0 | 0 | 2 | 80%+ | 0\13 | Ductal | 14.94 |
| 17# | 54 | 4 | 3 | III | 0 | 0 | 0 | 50%+ | 13\39 | Ductal | 7.77 |
| 18# | 43 | 3 | 2 | II | 2 | 3 | 1 | 10%+ | 1\19 | Ductal | 2.78 |
| 19# | 61 | 4 | 3 | II | 0 | 0 | 2 | 70%+ | 0\13 | Ductal | 6.43 |
| 20# | 79 | 6 | 2 | III | 3 | 3 | 3 | 10%+ | 5\13 | Ductal | 4.41 |

**Table S2** continued

| **Patient No.** | **Age(year)** | **Tumor size(cm)** | **Grade** | **TNM** | **ER** | **PR** | **HER2** | **MIB-1** | **Metastasis node count** | **Histological subtype** | **Fold repression of miR-133b(log_2_)** |
| --- | --- | --- | --- | --- | --- | --- | --- | --- | --- | --- | --- |
| 21# | 38 | 3 | 3 | III | 3 | 2 | 2 | 70%+ | 22\25 | Lobular and colloid | 12.71 |
| 22# | 58 | 3.5 | 3 | II | 0 | 0 | 3 | 30%+ | 0\14 | Ductal | 12.04 |
| 23# | 53 | 2 | 2 | I | 3 | 3 | 1 | 10%+ | 0\14 | Ductal | 8.03 |
| 24# | 81 | 4 | 3 | II | 0 | 0 | 2 | 80%+ | 0\16 | Ductal | 25.12 |
| 25# | 57 | 2.5 | 3 | II | 0 | 0 | 0 | 85%+ | 0\17 | Ductal | 10.71 |
| 26# | 37 | 3 | 2 | II | 1 | 1 | 3 | 30%+ | 2\13 | Ductal | -1.85 |
| 27# | 53 | 3.5 | 2 | II | 3 | 3 | 0 | 20%+ | 0\19 | Ductal | 32.80 |
| 28# | 56 | 5 | 3 | II | 1 | 0 | 2 | 30%+ | 0\17 | Medullary | 10.74 |
| 29# | 46 | 2.5 | 3 | II | 0 | 0 | 2 | 70%+ | 0\21 | Ductal and Medullary | 10.58 |
| 30# | 50 | 3 | 3 | II | 0 | 0 | 3 | 15%+ | — | Ductal | 3.71 |
| 31# | 48 | 2.5 | 3 | II | 2 | 1 | 3 | 15%+ | 0\4 | Ductal | 6.95 |
| 32# | 49 | 3.5 | 2 | II | 3 | 2 | 1 | 10%+ | 1\21 | Ductal | 11.35 |
| 33# | 54 | 2.5 | 3 | II | 0 | 0 | 3 | 25%+ | 0\11 | Ductal | 1.18 |
| 34# | 45 | — | 3 | II | 3 | 2 | 1 | 40%+ | — | Ductal | 16.17 |
| 35# | 38 | 3.5 | 2 | II | 1 | 2 | 3 | 45%+ | 5\17 | Ductal | 0.73 |
| 36# | 66 | 1 | - | II | 3 | 2 | 1 | 10%+ | 3\9 | Ductal | 18.82 |
| 37# | 72 | 0.5 | 3 | II | 3 | 3 | 3 | 20%+ | 0\10 | Ductal | 11.52 |
| 38# | - | - | - | - | - | - | - | - | - | - | 4.09 |

Note: ER, estrogen receptor; PR, progesterone receptor; For the values of ER, PR and HER2, 3 stands for strongly positive, 1 and 2 stand for mildly

positive, 0 stands for negative; - means missing data.
