# Supplementary material for: MiR-133b targets Sox9 to control pathogenesis and metastasis of breast cancer
Source: Cell Death Dis. 2018 Jul 3;9(7):752. doi: 10.1038/s41419-018-0715-6 (PMC6030174; doi:10.1038/s41419-018-0715-6)
Supplement: Supplementary file 3 — Table S3 [file 41419_2018_715_MOESM3_ESM.docx]

| **Table S3.** Characteristics and miR-133b expression in breast cancer patients. | | | |
| --- | --- | --- | --- |
| **Factors** | **Patients**  **Number (%)** | **log_2_^(fold repression of miR-133b)^**  **(mean ± SD)** | **p - value** |
| **Age (year)** |  |  | 0.177^a^ |
| ≤50 | 14 (37.83%) | -8.78 ± 6.61 |  |
| >50 | 23 (62.17%) | -8.08 ± 8.57 |  |
| Missing data | 1 |  |  |
| **Tumor size (cm)** |  |  | 0.364^a^ |
| ≤2 | 9(23.68%) | -12.51 ± 5.48 |  |
| >2 | 27(71.05%) | -10.21 ± 8.73 |  |
| Missing data | 2 |  |  |
| **Nodal status** |  |  | 0.215^a^ |
| Positive | 17 (44.73%) | -9.01 ± 7.86 |  |
| Negative | 18 (47.36%) | -12.46 ± 8.27 |  |
| Missing data | 3 |  |  |
| **MIB**  ≤20%  >20%  Missing data  **Grade**  ΙΙ  ΙΙΙ  Missing data  **TNM Stage**  Ι  ΙΙ  ΙΙΙ  Missing data  **ER status**  Strongly positive  Mildly positive  Negative  Missing data  **PR status**  Strongly positive  Mildly positive  Negative  Missing data  **HER2 status**  Strongly positive  Mildly positive  Negative  Missing data | 17(44.73%)  20(52.63%)  1  16(42.10%)  18(47.36%)  4  4(10.52%)  22(57.89%)  6(15.79%)  6  13(34.21%)  10(26.31%)  14(36.84%)  1  7(18.42%)  10(26.31%)  20(52.63%)  1  13(34.21%)  21(55.26%)  3(7.89%)  1 | -10.35 ± 8.13  -11.42 ± 8.01  -6.83 ± 6.34  -14.99 ± 7.75  -12.29 ± 7.22  -10.85 ± 8.67  -12.47 ± 8.85  -11.58 ± 8.13  -7.51± 6.70  -12.78±8.41  -12.56± 9.55  -8.73 ± 7.49  -11.46 ± 7.83  -10.26± 9.10  -10.69± 7.46  -11.16 ± 8.11 | 0.691 ^a^  0.002^a^  0.842 ^b^  0.911 ^b^  0.839 ^b^  0.581 ^b^ |

Note: ER, estrogen receptor; PR, progesterone receptor; ^a^Independent-Samples T test; ^b^Jonckheere-Terpstra test.
