# Supplementary material for: MiR-133b targets Sox9 to control pathogenesis and metastasis of breast cancer
Source: Cell Death Dis. 2018 Jul 3;9(7):752. doi: 10.1038/s41419-018-0715-6 (PMC6030174; doi:10.1038/s41419-018-0715-6)
Supplement: Supplementary file 4 — supplemental legends [file 41419_2018_715_MOESM4_ESM.docx]

**Legends for Supplemental Figures**

***Figure S1. Representative images of in situ hybridization with miR-133b LAN probe in breast tissue microarray assay.*** In situ hybridization of miR-133b in breast cancer tissues and adjacent normal breast tissues on a TMA which included 24 human samples of breast cancer (scale bars, 100 μm).Strong positive expression of miR-133b was observed in adjacent normal breast tissue while weakly positive expression of miR-133b in infiltrating ductal carcinoma. Scale bars, 100 μm.

***Figure S2. Overexpression of miR-133b suppresses proliferation of MDA-MB-231 and BT549 cells while has no effects on cell viability.* (**A) miR-133b overexpression suppresses proliferation of MDA-MB-231 and BT549 cells. MDA-MB-231 and BT549 cells transfected with 100 nM miR-133b and NC mimics were seeded in 96 well plates. CCK-8 assay was performed at 24, 48, 72 and 96 hr. (B, C) Overexpression of miR-133b has no effects on viability of MDA-MB-231 and BT549 cells. MDA-MB-231 and BT549 cells transfected with 100nM miR-133b and NC mimics were seeded in 6 well plates. After typsinization, cell viability was assessed by Vi-Cell XR Cell Viability Analyzer (Beckman Coulter) every two days (B). Phase-contrast images of MDA-MB-231 and BT549 at 4 and 6 days post transfection also shown that miR-133b does not induce obvious cell apoptosis (C). The symbols * denotes statistical difference (p<0.05) while ** and *** represent great significant difference (p<0.01 and p<0.001) by a two-tailed Student’s *t*-test. Scale bars, 100 μm.

***Figure S3. Ectopic overexpression of miR-133b suppresses colonigenic ability and metastasis-relevant traits of MDA-MB-231-luc cells in vitro.*** (A) The expression level of mature miR-133b in MDA-231-luc cells infected with plvx-miR-133b was measured by stem-loop qRT-PCR. (B) Effect of miR-133b on the proliferation of MDA-MB-231-luc cells. (C) Influence of miR-133b on colony formation of MDA-MB-231-luc cells. Representative dishes are presented (left panel). The number of clones formed was quantifiedand shown in the right panel. (D) Anchorage-independent growth assays of MDA-MB-231-luc cells infected with plvx-miR-133b or plvx-vector. MDA-MB-231-luc cells were cultured in soft agar medium for 21 days and formed colonies containing more than 50 cells per well were quantified. (E) Migration and Invasion assays of MDA-MB-231-luc cells infected with plvx-miR-133b or plvx-vector. Cell migration and invasion of above cells were quantitatively analyzed 18 hr after seeding in Transwells. Data are presented as mean ± SEM. The symbols ** and *** represent great significant difference (p<0.01 and p<0.001) by a two-tailed Student’s *t*-test. Scale bars, 100 μm.

***Figure S4. WAVE2 and Sox9 knockdowns phenocopy miR-133b-mediated phenotypes in BT549 cells.*** (A) Detection of endogenous WAVE2 and Sox9 expression at 48h after transfection of siWAVE2 or siSox9 in BT549 cells. β-Actin serves as an internal control. (B) Effects of transfection of siWAVE2 or siSox9 on the proliferation of BT549 cells. (C) Influence of siWAVE2 or siSox9 on colony formation of BT549 cells. Left panel, representative dishes by colony formation assay. Right panel, the number of colonies formed was quantified and normalized against the control. (D) Transwell migration and invasion assays of BT549 cells transfected with siWAVE2 or siSox9. Data are presented as mean ± SEM. The symbol * denotes statistical difference (p<0.05) while ** represents great significant difference (p<0.01) in a two-tailed Student’s *t*-test.

***Figure S5. Overexpression of WAVE2 partially reverses miR-133b-induced inhibition of invasion in MDA-MB-231-luc and BT549 cells in vitro.***(A) Expression of WAVE2 in MDA-MB-231-luc and BT549 cells co-transfected with NC or miR-133b mimics together with either pcDNA3.1(-)-vector or pcDNA3.1(-)-WAVE2 for 48 hr. (B) Invasion assays with MDA-MB-231-luc and BT549 cells co-transfected with NC or miR-133b mimics together with pcDNA3.1(-)-vector or pcDNA3.1(-)-WAVE2. Overexpression of WAVE2 impaired the reduction of invasion ability caused by overexpression of miR-133b in MDA-MB-231-luc and BT549 cells. Data are presented as mean ± SEM. The symbols * denotes statistical difference (p<0.05), whereas **represent great significant difference (p<0.01 and p<0.001) by a two-tailed Student’s *t*-test.

***Figure S6. Re-expression of Sox9 rescues miR-133b-induced inhibition of colonigenic ability in MDA-MB-231-luc cells in vitro.*** (A) Expression of Sox9 in miR-133b-expressing or control MDA-MB-231-luc cellsinfected with pMSCV-Sox9 or control vector. (B) In *vitro* growth corves of miR-133b-expressing or control MDA-MB-231-luc cells infected with pMSCV-Sox9 or control vector. (C) Colony formation assays for miR-133b-expressing or control MDA-MB-231-luc cells infected with pMSCV-Sox9 or control vector. Representative dishes are presented (Left panel). The number of clones was counted for each well of six plates and shown in the *y*-axis of the right panel. Data are presented as mean ± SEM. The symbols*** represent great significant difference (p<0.001) by a two-tailed Student’s *t*-test.

***Figure S7. Kaplan-Meier plots of breast cancer patients stratified by the expression levels of miR-133b.*** （n_high_=329, n_low_=933, log-rank test）MiRpower, a web-tool to validate survival-associated miRNAs utilizing expression data from breast cancer patients were utilized to do the Kaplan-Meier analysis. The METABRIC dataset were chosen because of it has long follow-up (median: 94 months), average characteristics (78% ER positive, 12% HER2 positive) and the largest number of breast cancer patients (1262 patients). Kaplan-Meier analysis showed that breast cancer patients with miR-133b-low tumours had significantly worse overall survival that those with miR-133b-high tumours.
