# Supplementary figures and images for: MiR-133b targets Sox9 to control pathogenesis and metastasis of breast cancer
Source: Cell Death Dis. 2018 Jul 3;9(7):752. doi: 10.1038/s41419-018-0715-6 (PMC6030174; doi:10.1038/s41419-018-0715-6)

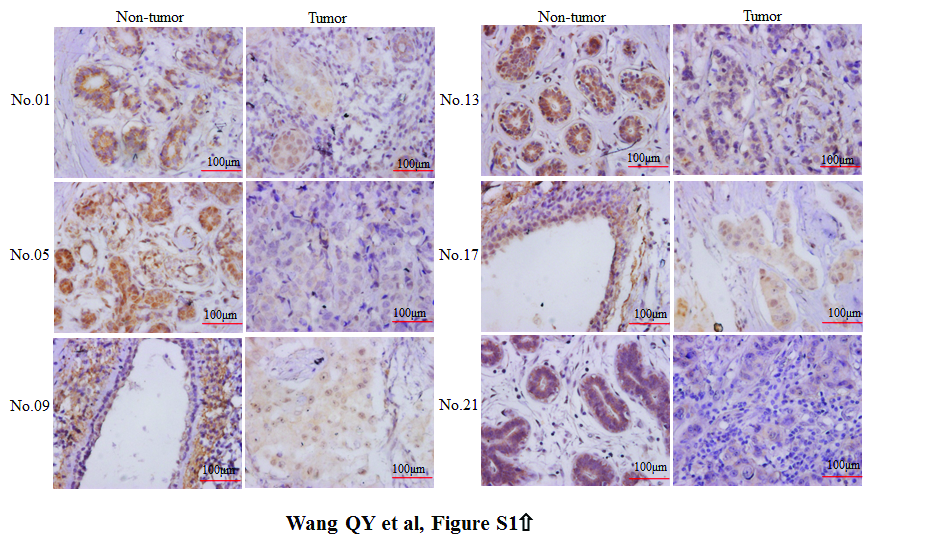

Supplement: Supplementary file 5 — Figure S1 [file 41419_2018_715_MOESM5_ESM.tif]

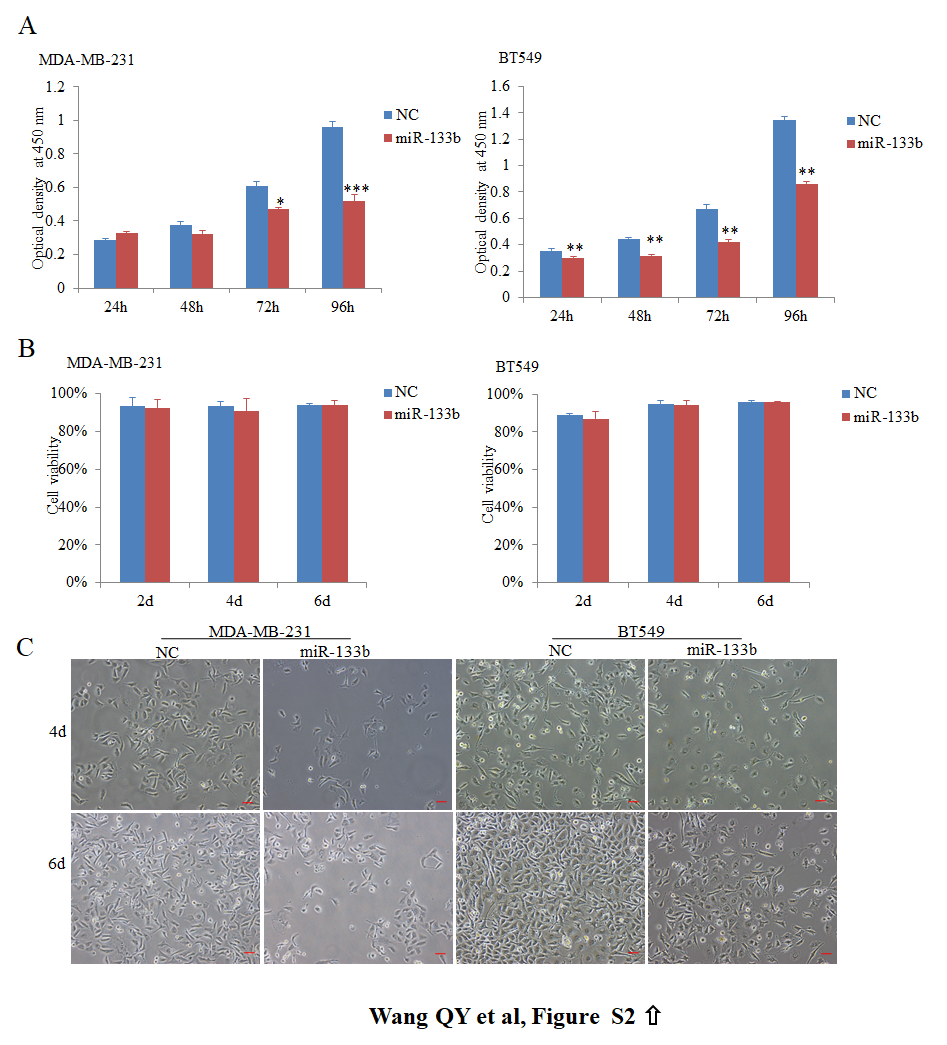

Supplement: Supplementary file 6 — Figure S2 [file 41419_2018_715_MOESM6_ESM.tif]

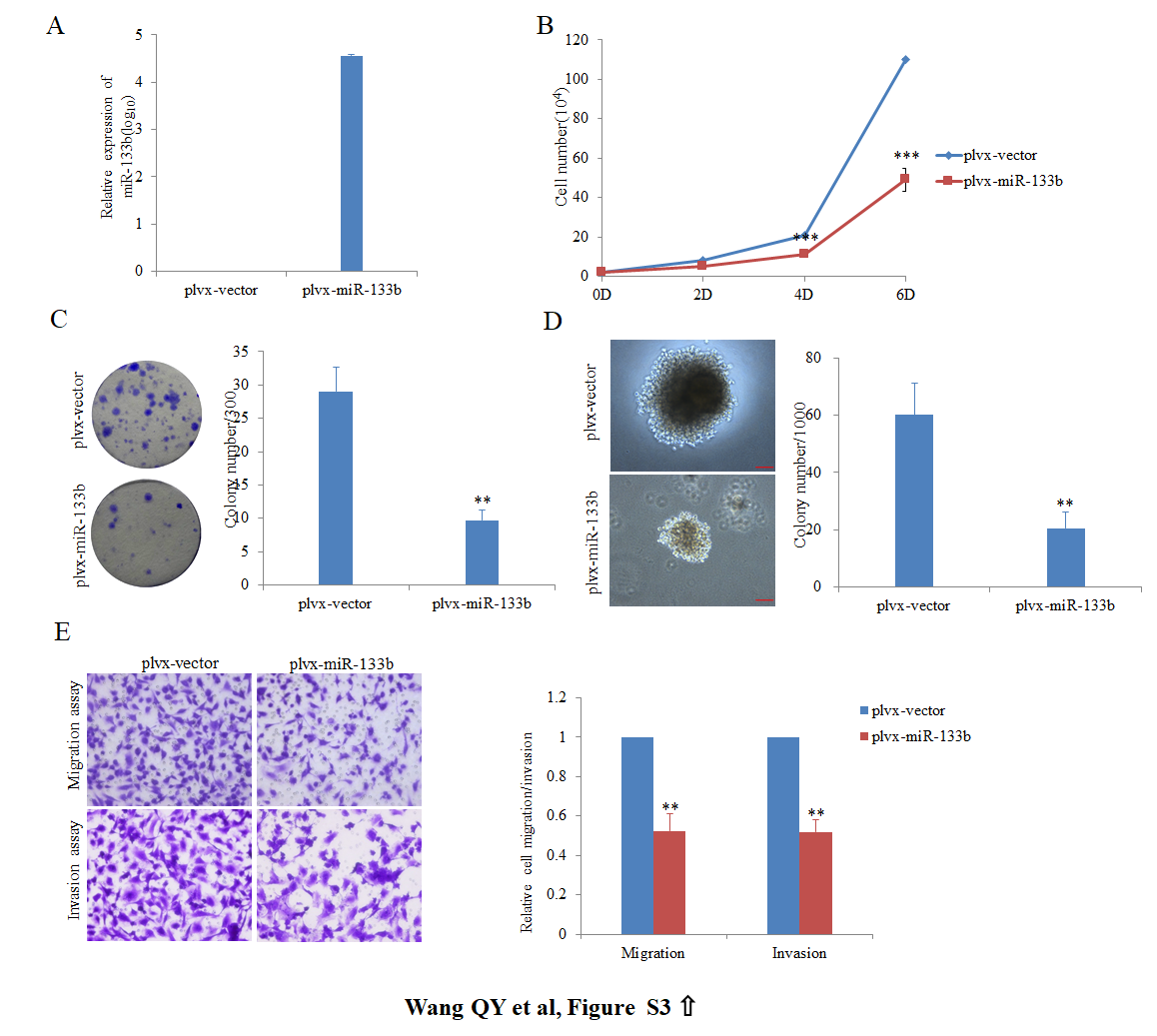

Supplement: Supplementary file 7 — Figure S3 [file 41419_2018_715_MOESM7_ESM.tif]

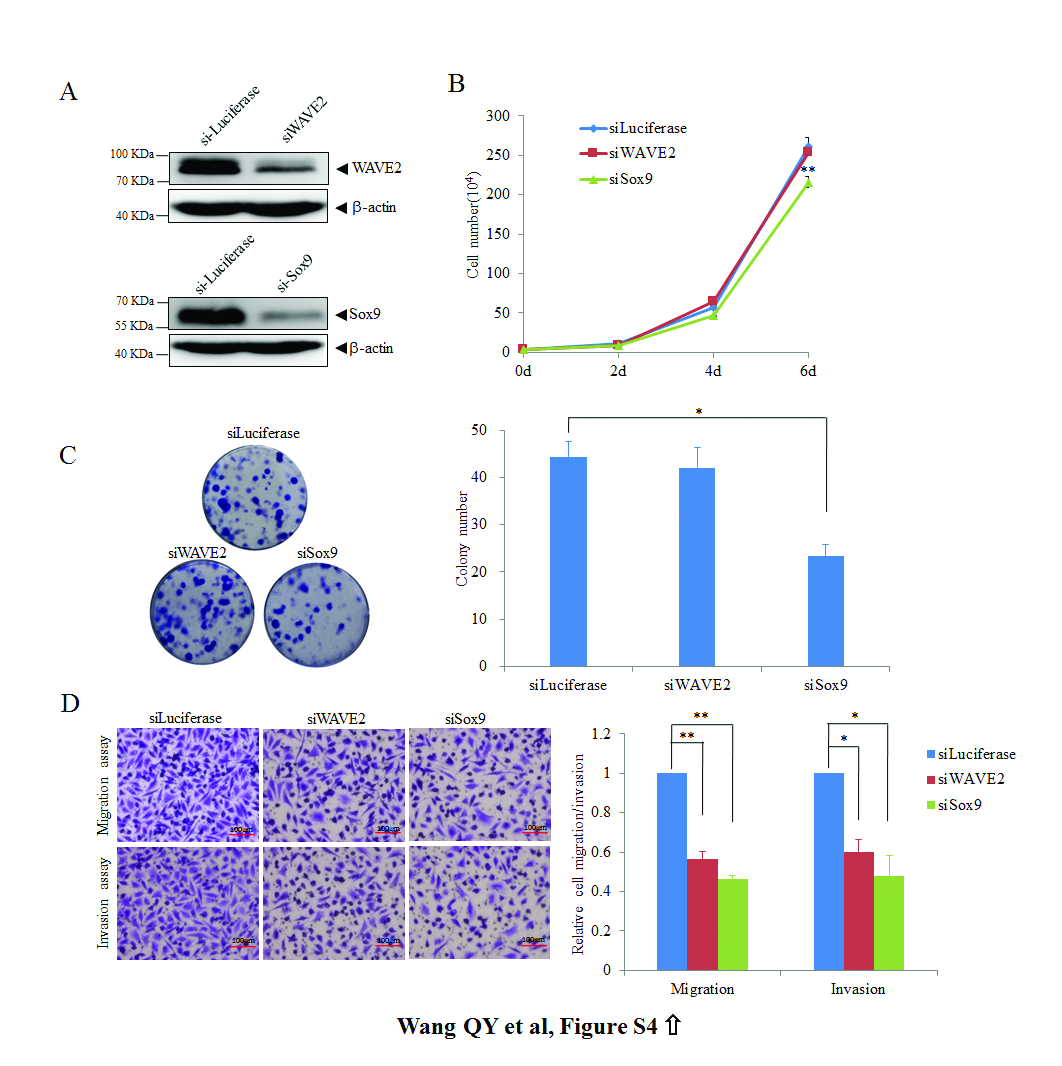

Supplement: Supplementary file 8 — Figure S4 [file 41419_2018_715_MOESM8_ESM.tif]

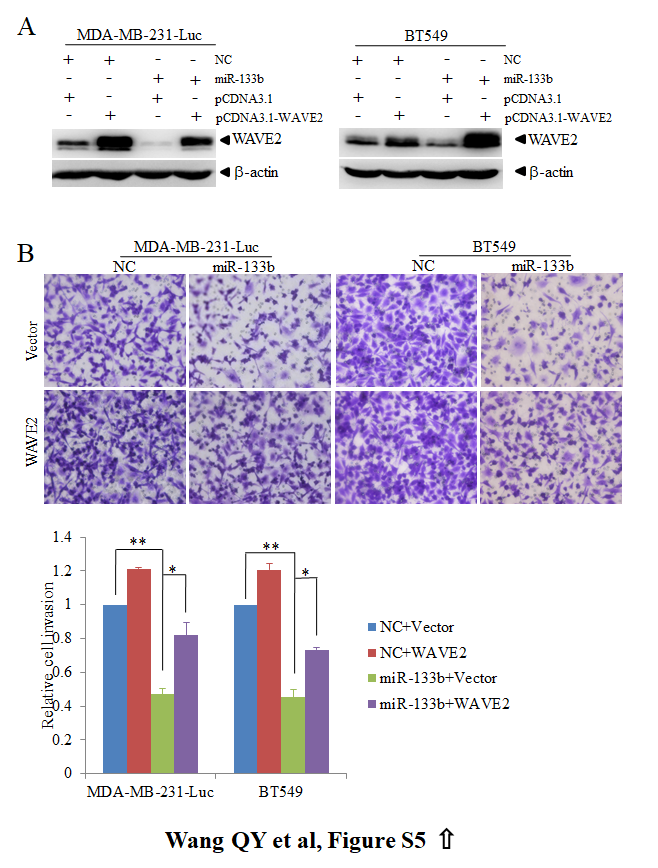

Supplement: Supplementary file 9 — Figure S5 [file 41419_2018_715_MOESM9_ESM.tif]

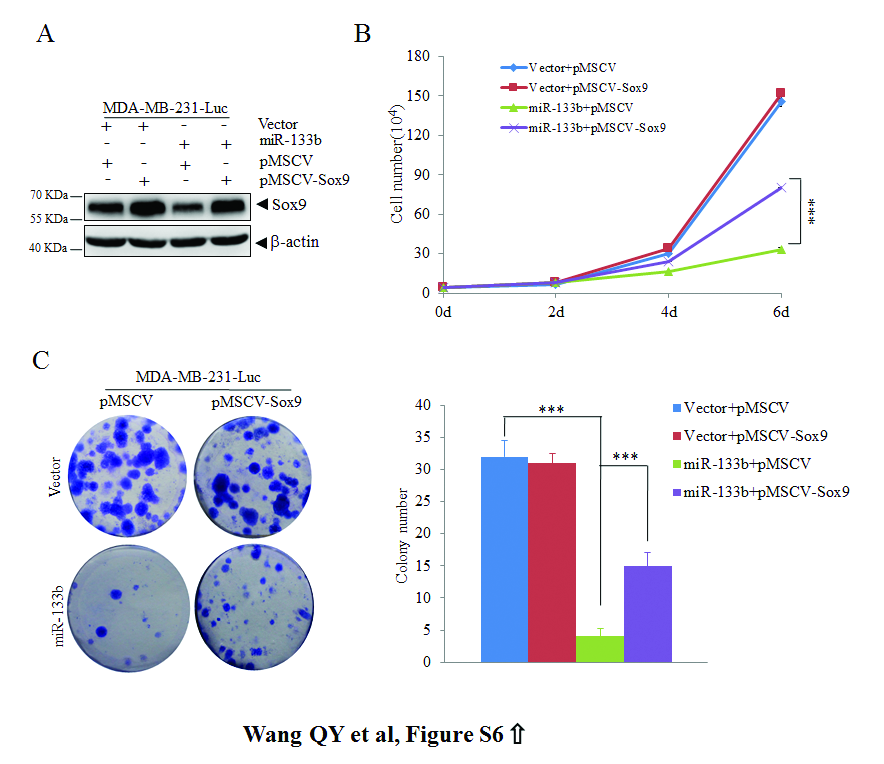

Supplement: Supplementary file 10 — Figure S6 [file 41419_2018_715_MOESM10_ESM.tif]

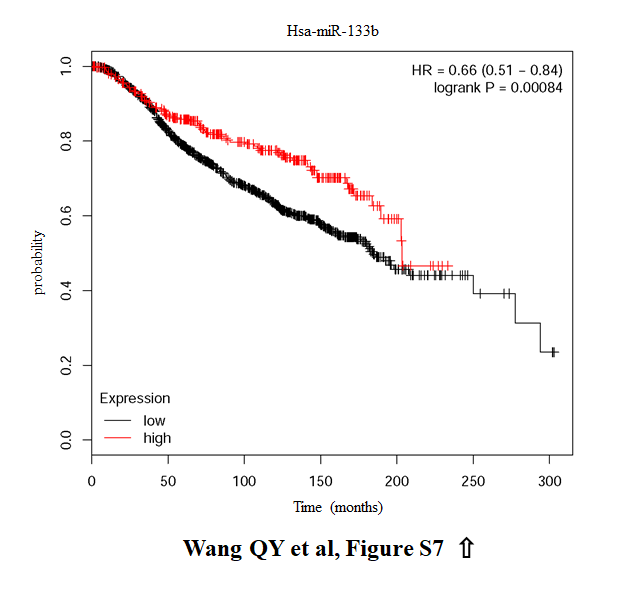

Supplement: Supplementary file 11 — Figure S7 [file 41419_2018_715_MOESM11_ESM.tif]
